# TROP2-targeted NIR-II fluorescence imaging for visualizing surgical margins and metastatic sentinel lymph nodes in breast cancers

**DOI:** 10.1073/pnas.2519420123

**Published:** 2026-02-19

**Authors:** Kang-Liang Lou, Jing-Wen Bai, Hong-Tan Liu, Lin-Ling Lin, Cheng-Xi Li, Yi-Yang Gao, Sheng-Jie Lin, Yi-Fei Pei, Xiao-Long Wei, Yi-Xin Chen, Yi-Yin Tang, Hong-Yu Chen, Zhi-Yao Li, Rong Guo, Shi-Cong Tang, Chuan-Liu Wu, Guo-Jun Zhang

**Affiliations:** ^a^Fujian Key Laboratory of Precision Diagnosis and Treatment in Breast Cancer and Xiamen Key Laboratory of Endocrine-Related Cancer Precision Medicine, School of Medicine, Xiamen University, Xiamen, Fujian 361102, China; ^b^The Breast Center and the Cancer Institute of Yunnan Cancer Hospital, Yunnan Key Laboratory of Molecular Imaging in Oncology, The Third Affiliated Hospital of Kunming Medical University, Yunnan Cancer Hospital, Peking University Cancer Hospital Yunnan, Kunming, Yunnan 650118, China; ^c^Department of Rehabilitation and Palliative Medicine, The Third Affiliated Hospital of Kunming Medical University, Yunnan Cancer Hospital, Peking University Cancer Hospital Yunnan, Kunming, Yunnan 650118, China; ^d^Ministry of Education Key Laboratory of Spectrochemical Analysis and Instrumentation, Department of Chemistry, College of Chemistry and Chemical Engineering, Xiamen University, Xiamen, Fujian 361005, China; ^e^Department of Pathology, Cancer Hospital of Shantou University Medical College, Shantou, Guangdong 515041, China; ^f^Department of Medical Ultrasound, The Third Affiliated Hospital of Kunming Medical University, Yunnan Cancer Hospital, Peking University Cancer Hospital Yunnan, Kunming, Yunnan 650118, China

**Keywords:** breast cancer, surgical margin, sentinel lymph node, NIR-II fluorescence imaging–guided surgery, TROP2

## Abstract

In early breast cancer surgery (EBC), free margins significantly decrease local recurrence in breast-conserving surgery (BCS), while metastatic status of sentinel lymph node (SLN) impacts axillary management. However, intraoperative visualization of margins and metastatic SLNs (mSLNs) remains challenging. Here, we report a second-window near-infrared (NIR-II) fluorescence probe by conjugating ICG to TROP2-targeting cyclic peptide (TTP-ICG). Our results showed that TTP-ICG specifically binds to TROP2-expressing cells in vitro and identifies TROP2-positive tumors in vivo. In addition, TTP-ICG enabled efficient intraoperative evaluation of surgical margins and visualization of mSLNs at a submillimeter level in preclinical models. Additionally, we optimized the rapid incubation imaging method (RIIM) by shortening the procedure to less than 8 min, illustrating TTP-ICG’s high performance in distinguishing malignant from normal tissues/fibroadenoma lesions as well as detecting metastatic lymph nodes in a cohort of 59 patients. Thus, this TROP2-targeting probe demonstrates its significance for fluorescence imaging-guided surgery with dual applicability in EBC, providing translational potential for further clinical decision-making.

## Significance

This study proposes a solution to the critical challenge of rapid and accurate intraoperative assessment of surgical margins and metastatic lymph nodes during early breast cancer surgery. We introduce a TROP2-targeted near-infrared (NIR-II) fluorescence probe, TROP2-targeting cyclic peptide (TTP)-ICG, which demonstrated high specificity for TROP2-expressing tumors and metastatic lymph nodes in preclinical models. Meanwhile, the TTP-ICG-based rapid incubation imaging method (RIIM) provides assessment results within 8 min, accurately distinguishing malignant from normal/fibroadenoma tissues and identifying metastatic lymph nodes in a cohort of 59 patients. This dual-capability technology offers transformative potential for fluorescence-guided surgery, improving oncologic outcomes by reducing positive margins and unnecessary lymph node procedures, thereby advancing precision breast cancer surgery.

Breast cancer (BC) is the most prevalent malignancy among women globally, posing a serious threat to women’s health (1, 2). For early BC (EBC) patients, breast-conserving surgery (BCS) and sentinel lymph node biopsy (SLNB) have emerged as the cornerstone of precision oncology, harmonizing oncologic efficacy with the preservation of breast cosmetics and axillary function (3–5). BCS aims to achieve tumor-free margins to minimize local recurrence, while SLNB provides critical axillary staging information to guide adjuvant therapy decisions—two interdependent components that collectively define surgical success. Notably, in clinical practice, about 60% of BCS procedures are performed alongside SLNB (6–8), which not only guarantee thorough tumor excision and precise LN staging but also preserve breast aesthetics and minimize the complications associated with axillary lymph node dissection (ALND).

However, current intraoperative workflows for BCS and SLNB face interrelated limitations that compromise their efficacy. During BCS, the status of margin is critically related to postoperative local recurrence, as both adjuvant radiotherapy and systemic therapy offer limited compensation on recurrence risk when positive margins occur (9). However, margin assessment heavily relies on surgeons’ subjective experience, leading to positive margins as high as 15 to 50% (10, 11), which necessitate re-excision and increase physical and economic burdens. Although current intraoperative assessment techniques, including frozen section (12) and touch imprint cytology (13), have reduced positive margin rates significantly, they remain constrained by procedural complexity, time-consumption, and imperfect accuracy.

As SLNs are the pivotal predictor of non-SLN involvement, the technology to accurately identify SLN metastases intraoperatively would allow node-negative patients to be exempt from ALND and related complications. For this purpose, SLNB has emerged as a reliable and mini-invasive alternative to ALND for clinically node-negative patients’ stratification (14, 15). However, similar with the margin assessment, intraoperative evaluation of SLN’s metastatic status mainly depends on frozen pathology, which is laborious with relatively high false-negative rates, and is particularly difficult to detect micrometastases (16–18). Moreover, about 70 to 80% of patients receiving SLNB are ultimately confirmed with negative nodes, while a recent study reports an extremely low nodal positivity rate of 6% at single-facility level (19), suggesting that a significant proportion of patients might be omitted from invasive procedures to avoid unnecessary complications such as lymphedema and sensory deficits (20). This concept is recently further supported by the results from two randomized clinical trials (SOUND and INSEMA), which illustrated the feasibility of omitting axillary surgery in highly selected cases with ultrasound-diagnosed negative nodes (21, 22). Therefore, the capacity for rapid, intraoperative identification of SLN metastases would not only enable surgeons to proceed directly to ALND when SLN involvement is detected instead of waiting for pathology but also spare node-negative patients from SLNB and related complications, thereby significantly reducing the overall physical and emotional burden on patients. These findings prompted researchers to explore technologies that enable rapid and precise evaluation of both margins and nodal status to refine surgical decision-making.

Fluorescence imaging in the second near-infrared window (NIR-II, 1,000 to 1,700 nm) with indocyanine green (ICG) has been shown to significantly reduce the light absorption, scattering, and tissue autofluorescence, thereby enhancing penetration depth, imaging contrast, and resolution (23–25), and seems suitable to address these challenges via a transformative approach. For example, ICG alone could exhibit excellent in vivo imaging performance in guiding oral cancer (26), liver tumor (27), and cystic renal masses excision (28). However, ICG solely relies on the enhanced permeability and retention (EPR) effect for tumor recognition (29), which limits its specificity and accuracy. Accordingly, introducing a specific tumor-targeted moiety holds promise for achieving more precise and efficient intraoperative guidance in BCS and SLN metastasis status assessment (30, 31).

Trophoblast cell surface antigen 2 (TROP2) is highly expressed in BC but is almost not expressed or absent in normal breast tissues, and therefore might represent an ideal molecular target (32–34). Emerging TROP2-directed imaging agents were already developed for BC visualization (35–39) by either nuclide or optical dye labeling. In addition to antibodies and small molecules, peptide-based probes, with their intermediate size and sufficient binding affinity, strike a balance between antibody-mediated specificity and small molecule’s penetration, minimizing immunogenicity while enhancing tumor retention (40–42). In an attempt to obtain such peptides specific to TROP2, we screened out a TROP2-targeting cyclic peptide (TTP) via phage display technology in CPPC-paired disulfide-rich peptide libraries as previously reported (43). The peptide was then covalently conjugated to ICG to generate the NIR-II fluorescent probe TTP-ICG.

In this study, we demonstrate TTP-ICG’s dual capability to in vivo delineate tumor margins and identify metastatic SLNs (mSLNs) in preclinical models, supported by an ex vivo rapid incubation imaging method (RIIM) validated in 48 BC and 11 fibroadenoma patients (NCT06713681) (*SI Appendix*, Fig. S1). This integrated approach overcomes current technical limitations by providing molecular-specific intraoperative guidance, representing an advancement toward precise breast cancer surgery.

## Results

### Successful Synthesis and Characterization of TTP-ICG.

Following conjugation with ICG via NHS ester chemistry, the TTP-ICG displayed a slight red shift in both absorption and emission spectra compared to free ICG, while maintaining strong 808 nm absorption and NIR-II tailing-emission properties (*SI Appendix*, Fig. S2 *A*–*C*). High-performance liquid chromatography (HPLC) analysis under gradient elution (0.1% TFA/water [solvent A] and acetonitrile [solvent B]; 15 to 90% B over 40 min, 1 mL/min) confirmed the successful synthesis, with distinct retention times observed for TTP, ICG-NHS, and the crude TTP-ICG mixture. Subsequent purification yielded TTP-ICG with >90% purity (*SI Appendix*, Fig. S2*D*). Mass spectrometry further corroborated the molecular weights of TTP (3,331.008 Da) and TTP-ICG (4,044.123 Da), aligning precisely with theoretical values (*SI Appendix*, Fig. S2*E*).

### TTP-ICG Demonstrates High TROP2-Specific Targeting in BC Cells.

As depicted in *SI Appendix*, Fig. S3*A*, MCF-7 cells exhibited the highest TROP2 expression among the evaluated BC cell lines, while 4T1-Luc and MDA-MB-231-Luc displayed relatively lower levels. To validate TROP2 targeting, we generated TROP2-overexpressing cell lines (MDA-MB-231-Luc-TROP2 and 4T1-Luc-TROP2) and a TROP2-knockdown cell line (MCF-7-shTROP2) using plasmid or lentiviral transfection (*SI Appendix*, Fig. S3 *B*–*D*).

**Fig. 1.**
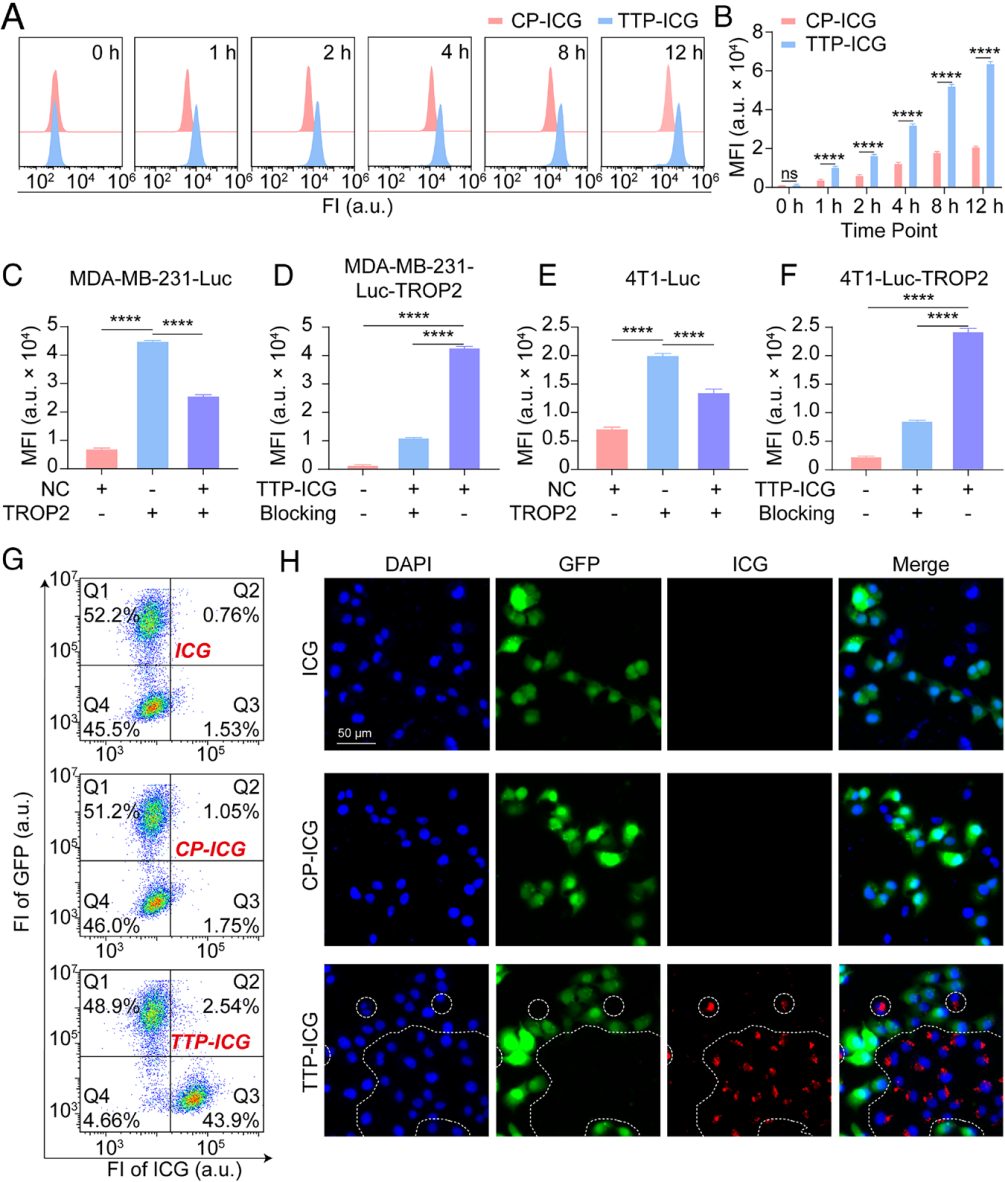
Validation of TTP-ICG’s targetability to TROP2 in cell lines. (*A* and *B*) FCM analysis (*A*) and MFI of MDA-MB-231-Luc-TROP2 cells (*B*) at different time points after TTP-ICG/CP-ICG incubation. (*C*) Cellular MFIs measured by FCM after incubating TTP-ICG with MDA-MB-231-Luc-NC, MDA-MB-231-Luc-TROP2, and a 1:1 mixture of both cell lines. (*D*) MFI of MDA-MB-231-Luc-TROP2 cells measured by FCM after 8-h incubation with PBS, TTP-ICG, or TTP-ICG preblocked with 40-fold excess TTP. (*E*) Cellular MFIs measured by FCM after incubating TTP-ICG with 4T1-Luc-NC, 4T1-Luc-TROP2, and a 1:1 mixture of both cell lines. (*F*) MFI of 4T1-Luc-TROP2 cells measured by FCM after 8-h incubation with PBS, TTP-ICG, or TTP-ICG preblocked with 40-fold excess TTP. (*G* and *H*) FCM analysis (*G*) and representative fluorescence microscopy images (*H*) after incubating 1:1 mixed system of MDA-MB-231-Luc-GFP and MDA-MB-231-Luc-TROP2 cells with ICG, CP-ICG, or TTP-ICG. (****P < 0.0001, mean ± SD, n = 3).

Treatment with either the control probe (CP-ICG) or TTP-ICG induced time-dependent increases in mean fluorescence intensity (MFI) in MDA-MB-231-Luc-TROP2 cells (Fig. 1*A*). Notably, the MFI in the TTP-ICG-treated group was significantly higher than that in the CP-ICG group (*P* < 0.0001). Meanwhile, at 8 h posttreatment, TTP-ICG-treated cells demonstrated a ~2.88-fold increase in MFI compared to CP-ICG (*P* < 0.0001; Fig. 1*B*).

To evaluate TROP2-dependent uptake, TTP-ICG internalization was compared across cell lines with varying TROP2 expression. Following an 8-h incubation, MDA-MB-231-Luc-TROP2 (high-TROP2) exhibited significantly higher MFI than MDA-MB-231-Luc-NC (low-TROP2) and a 1:1 mixed population (7,140.67 ± 174.43 vs. 25,751.00 ± 299.56 vs. 45,053.33 ± 106.52; *P* < 0.0001; Fig. 1*C*). Odyssey® CLX imaging corroborated these findings, showing markedly stronger fluorescence in TROP2-overexpressing cells (*SI Appendix*, Fig. S4*A*). Meanwhile, competitive inhibition with 40-fold excess free TTP reduced TTP-ICG internalization by ~74% (*P* < 0.0001; Fig. 1*D*).

This TROP2-dependent uptake pattern was consistently observed across multiple cell systems. In the 4T1-Luc cell system, 4T1-Luc-TROP2 cells (high-TROP2) demonstrated a higher MFI than 4T1-Luc-NC (low-TROP2) and the mixed population under Odyssey® CLX imaging (*SI Appendix*, Fig. S4*B*), flow cytometry (FCM; *P* < 0.0001; Fig. 1*E*), and fluorescence microscopy (*SI Appendix*, Fig. S4*D*). Excess TTP suppressed uptake by ~65% (*P* < 0.0001; Fig. 1*F*). In the MCF-7 cell system, MCF-7-shNC (high-TROP2) also showed superior MFI than MCF-7-shTROP2 (low-TROP2) and the mixed population (*SI Appendix*, Fig. S4 *C*, *E*, and *F*), collectively confirming a robust correlation between TROP2 expression and TTP-ICG uptake.

To further validate TTP-ICG’s TROP2-targeting efficacy, a 1:1 coculture system was established using MDA-MB-231-Luc-GFP (low-TROP2/high-GFP) and MDA-MB-231-Luc-TROP2 (high-TROP2/GFP-negative) cells. Following an 8-h TTP-ICG incubation, FCM revealed distinct cellular populations: GFP-positive cells (low-TROP2) showed minimal ICG fluorescence, whereas GFP-negative cells (high-TROP2) exhibited robust ICG signals (*SI Appendix*, Fig. S4*G*). A ~5.6-fold higher ICG intensity was observed in the TROP2-high (GFP-low, Q3) population compared to the TROP2-low (GFP-high, Q1) population (*P* < 0.0001; *SI Appendix*, Fig. S4*H*). Similarly, as demonstrated in Fig. 1 *G* and *H*, TTP-ICG treatment resulted in clear separation of the two populations under the ICG channel in both FCM and microscopy. In contrast, treatment with nontargeted probes (ICG or CP-ICG) showed no significant fluorescence differences between cell clusters, whether analyzed by FCM or fluorescence microscopy, providing definitive evidence of TTP-ICG’s TROP2-specific targeting capability.

Additionally, hemolysis assays revealed excellent blood compatibility of TTP-ICG, with hemolysis rates remaining below 5% even at the highest tested concentration (800 nM; *SI Appendix*, Fig. S5 *A* and *B*). Furthermore, cell viability assays demonstrated satisfactory safety of TTP-ICG, with >95% viability maintained in both cancerous (MDA-MB-231-Luc, MCF-7) and normal (MCF-10A) breast cell lines at concentrations up to 800 nM (*SI Appendix*, Fig. S5*C*)

### TTP-ICG Achieves Superior Tumor Specificity In Vivo.

**Fig. 2.**
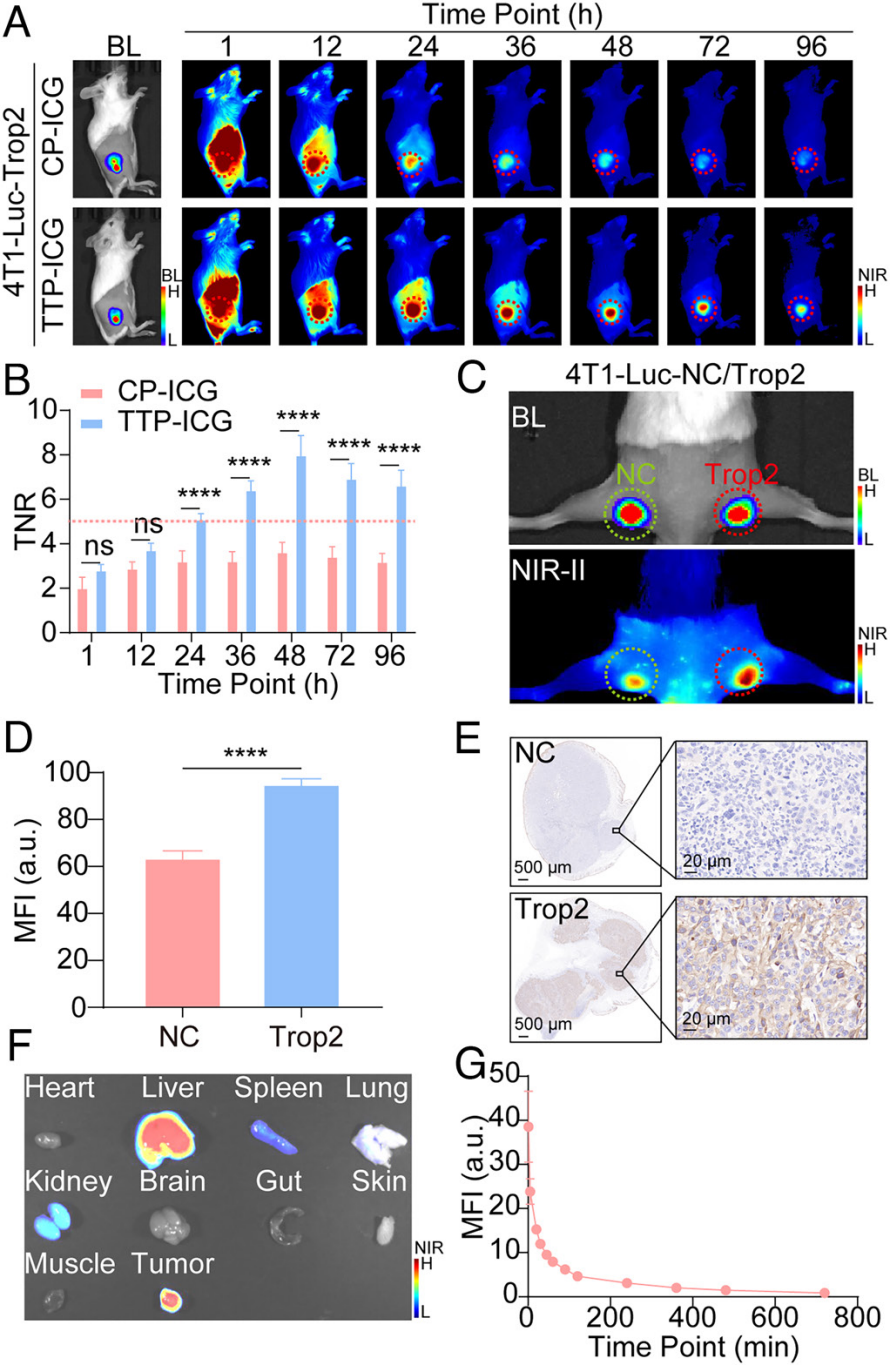
In vivo assessment of TTP-ICG’s targetability to TROP2 with NIR-II imaging. (*A* and *B*) Representative in vivo BL and NIR-II images (*A*) as well as TNR (*B*) analysis after intravenous injection of CP-ICG/TTP-ICG in 4T1-Luc-TROP2 subcutaneous tumor model. The red dotted line is according to the Rose criterion. (*C*–*E*) Representative BL and NIR-II image (*C*), tumor NIR-II MFI analysis (*D*) and TROP2-immunohistochemistry (IHC, *E*) at 48 h postinjection of TTP-ICG in bilateral tumor model of 4T1-Luc-NC and 4T1-Luc-TROP2. (*F*) Representative NIR-II image for biodistribution of TTP-ICG at 48 h postinjection in 4T1-Luc-TROP2 subcutaneous tumor model. (*G*) Decay curve of blood MFI after TTP-ICG intravenous administration (****P < 0.0001, mean ± SD, n = 4).

The tumor-targeted efficacy of TTP-ICG was further evaluated in murine models. In unilateral subcutaneous tumor models (4T1-Luc-TROP2 or MDA-MB-231-Luc-TROP2), intravenous TTP-ICG administration initially showed high background signals in the liver and blood circulation. With the clearance of TTP-ICG, mice demonstrated progressively increasing tumor-normal ratios (TNRs), peaking at 48 h postinjection (Fig. 2*A* and *SI Appendix*, Fig. S6*A*). Notably, TTP-ICG exhibited significantly higher TNRs compared to CP-ICG (4T1-Luc-TROP2: 8.00 ± 0.87 vs. 3.65 ± 0.41; MDA-MB-231-Luc-TROP2: 5.72 ± 0.98 vs. 2.60 ± 0.40; *P* < 0.0001), exceeding the Rose criterion (44) threshold (TNR > 5), which is required for a human observer to confidently identify a mass above background (Fig. 2*B* and *SI Appendix*, Fig. S6*B*). In bilateral subcutaneous tumor models with varying TROP2 expression, TTP-ICG accumulated preferentially in TROP2-high tumors (*P* < 0.0001, Fig. 2 *C*–*E*; *P* < 0.001, *SI Appendix*, Fig. S6 *C*–*E*). Frozen tumor-muscle sections further confirmed tumor-selective fluorescence (*SI Appendix*, Fig. S7).

Biodistribution analysis at 48 h revealed the highest MFI in the tumor and liver, with moderate signals in the kidney, lung, and spleen (Fig. 2*F* and *SI Appendix*, Fig. S8). Liver, lung, and spleen fluorescence correlated with the activity of the reticuloendothelial system, while kidney signals reflected the renal clearance of TTP-ICG. Moreover, pharmacokinetic studies confirmed rapid blood clearance (half-life (t_1/2_) = 15.57 min; Fig. 2*G*).

Additionally, TTP-ICG demonstrated no significant toxicity in BALB/c mice. Blood routine and biochemistry analyses showed no abnormalities at 1, 7, and 28 d posttreatment (*SI Appendix*, Fig. S9 *A* and *B*). Histopathology of major organs (heart, liver, spleen, lung, kidney, brain) and injection sites revealed no morphological or architectural changes (*SI Appendix*, Fig. S9 *C* and *D*).

### TTP-ICG Precisely Guided Tumor Resection in Various Mouse Models.

**Fig. 3.**
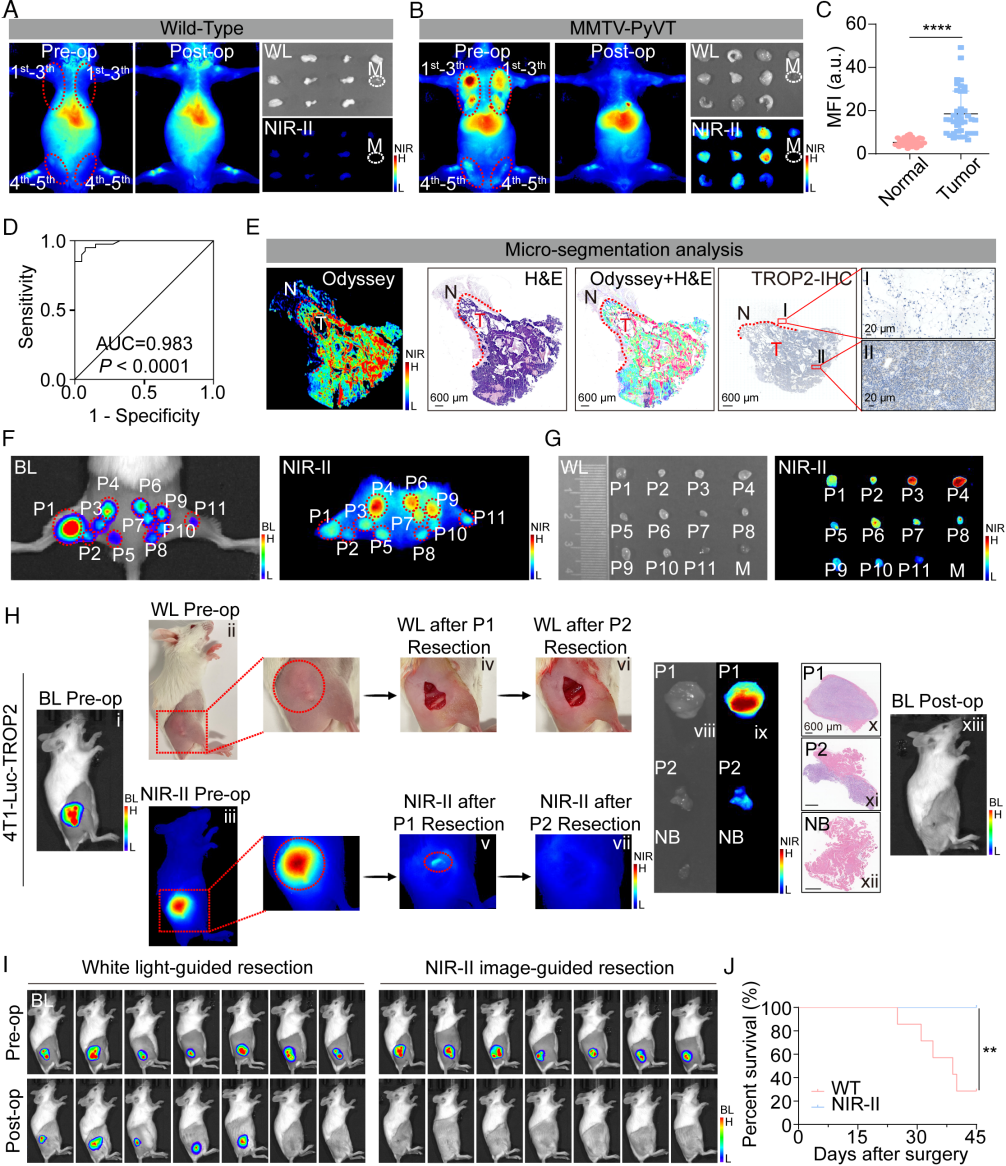
NIR-II fluorescence-guided tumor resection in mouse models. (*A* and *B*) Representative in vivo and ex vivo NIR-II images of wild-type mice (*A*, n = 4) and MMTV-PyVT mice (*B*, n = 4) before and after 5 pairs of mammary glands excision. (*C* and *D*) Fluorescence quantification (*C*), and ROC curve (*D*) of ex vivo mammary gland tissues according to the pathology results (n = 40). (*E*) Representative microsegmentation fluorescence and pathological analyses. (*F*) Representative BL and NIR-II images of multiple microtumors mouse model. (*G*) NIR-II images of tumors resected from (*F*). (*H*) Representative procedure for NIR-II fluorescence-guided resection in the 4T1-Luc-TROP2 intramuscular tumor-invasion model. All scale bars represent 600 μm. (*I* and *J*) Recurrence (*I*) and survival (*J*) after tumor resection in the 4T1-Luc-TROP2 intramuscular tumor-invasion model (n = 7) (**P < 0.01, ****P < 0.0001, mean ± SD).

To evaluate TTP-ICG’s diagnostic efficiency, MMTV-PyVT mice with spontaneous breast tumors were employed for NIR-II fluorescence imaging. Both in vivo and ex vivo imaging at 48 h postinjection revealed stronger fluorescence signals in malignant tissues from MMTV-PyVT mice than normal mammary glands from wild-type mice (Fig. 3 *A* and *B*). The association between TROP2 expression and TNR of in vivo NIR-II imaging was analyzed from 40 MMTV-PyVT tumors. Heterogeneous expression of TROP2 was demonstrated, with 15 of 40 tumors exhibiting TROP2-high level (H-Score 200-300) and 25 of 40 showing TROP2-moderate level (H-Score 100-200). Notably, the TROP2-high tumors exhibited a significantly higher TNR than the TROP2-moderate cases (9.67 ± 0.99 vs. 5.84 ± 2.21; *P* < 0.0001; *SI Appendix*, Fig. S10), with both groups exceeding the Rose criterion (TNR > 5). Moreover, according to the ex vivo NIR-II imaging, malignant tissues exhibited higher MFI than normal tissues and the receiver operating characteristic (ROC) curve analysis demonstrated exceptional diagnostic accuracy (AUC = 0.983; 95% CI: 0.962 to 1.000; Fig. 3 *C* and *D*). Furthermore, fluorescence-guided microsegmentation sharply delineated invasive carcinomas from adjacent normal tissue, coinciding with H&E and TROP2-IHC staining (Fig. 3*E* and *SI Appendix*, Fig. S11).

Moreover, in a multiple-microtumor model, TTP-ICG identified all 39 lesions (including tumors as small as 2 mm) under NIR-II imaging with 100% accuracy (Fig. 3 *F* and *G*). Subsequent NIR-II-guided resection resulted in complete tumor removal with pathologically confirmed clear margins (*SI Appendix*, Fig. S12).

Furthermore, in intramuscular tumor-invasion models with 4T1-Luc-TROP2 and MDA-MB-231-Luc-TROP2, TTP-ICG-based NIR-II imaging precisely delineated tumor margins and guided complete tumor resection (Fig. 3*H* and *SI Appendix*, Fig. S13*A*). Initial white-light resection (P1) was followed by NIR-II-guided removal of residual fluorescent tissue (P2), ensuring that no residual signals remained (iv–vii). Ex vivo imaging and histopathology confirmed greater fluorescence in tumor tissues (P1/P2) compared to normal tumor bed tissues (viii–xii), with colocalization of fluorescence, H&E, and TROP2 expression (*SI Appendix*, Fig. S13*B*). NIR-II guidance reduced recurrence rates (0% vs. 71 to 86% in white-light groups; Fig. 3*I* and *SI Appendix*, Fig. S13*C*) and enhanced survival (*P* < 0.0001; Fig. 3*J* and *SI Appendix*, Fig. S13*D*). Furthermore, in an intramuscular tumor-invasion model with ER+ cell line (MCF-7-Luc with high TROP2 expression), TTP-ICG enabled high-contrast in vivo NIR-II imaging (TNR ~ 7.30) and effectively guided the precise resection of these tumors (*SI Appendix*, Fig. S14). These demonstrated that TTP-ICG-based NIR-II imaging is a valuable tool for intraoperative margin assessment and complete tumor clearance.

### TTP-ICG Accurately Guided Assessment for Metastatic Status of SLNs in Various Mouse Models.

**Fig. 4.**
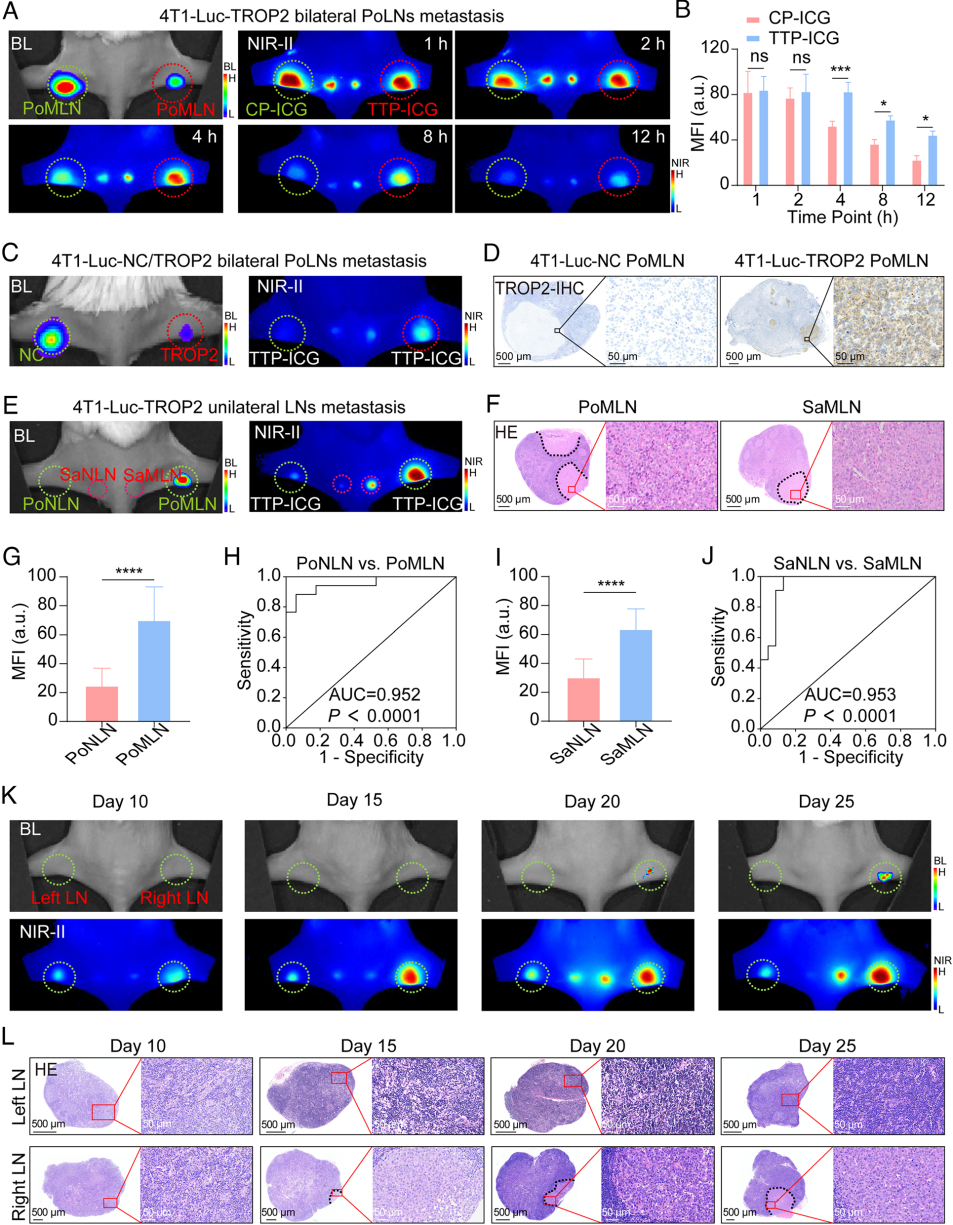
Visualization of metastatic SLNs in mouse model with NIR-II fluorescence-guidance. (*A* and *B*) Representative BL, NIR-II images (*A*), and NIR-II MFI analysis (*B*) of bilateral PoLNs after subcutaneous injection of CP-ICG/TTP-ICG via bilateral foot pads in 4T1-Luc-TROP2 bilateral PoLN metastasis model (n = 4). (*C* and *D*) Representative BL, NIR-II image (*C*), and TROP2-IHC (*D*) of bilateral PoLNs at 12 h postinjection of TTP-ICG in bilateral PoLN metastasis model with 4T1-Luc-NC and 4T1-Luc-TROP2 (n = 4). (*E*) Representative BL and NIR-II images of PoLNs and SaLNs at 4 h postinjection of TTP-ICG in 4T1-Luc-TROP2 unilateral LN metastasis model (n = 17). (*F*) H&E staining results of metastatic PoLN (PoMLN) and SaLN (SaMLN) from *E*. (*G* and *H*) NIR-II MFI analysis (*G*) and ROC curve (*H*) of nonmetastatic PoLNs (PoNLNs) and PoMLNs (n = 17 for each). (*I* and *J*) NIR-II MFI analysis (*I*) and ROC curve (*J*) of nonmetastatic SaLNs (SaNLNs, n = 23) and SaMLNs (n = 11). (*K*) Representative BL and NIR-II images of PoLNs at 4 h postinjection of TTP-ICG in mice with different time points (10, 15, 20, 25 d) after 4T1-Luc-TROP2 unilateral PoLN metastasis modeling. (*L*) H&E staining results of resected PoLNs from *K*. (*P < 0.05, ***P < 0.001, **** *P* < 0.0001, mean ± SD).

After subcutaneous injection of TTP-ICG or CP-ICG into the hind foot pads of 4T1-Luc-TROP2 or MDA-MB-231-Luc-TROP2 bilateral popliteal lymph node (PoLN) metastasis models, TTP-ICG-treated metastatic PoLNs (PoMLNs) showed significantly higher fluorescence accumulation than the controls, with maximal contrast achieved at 12 h (4T1-Luc-TROP2: 44.51 ± 3.32 vs. 22.69 ± 3.45; MDA-MB-231-Luc-TROP2: 27.26 ± 7.15 vs. 9.36 ± 0.84; *P* < 0.05; Fig. 4 *A* and *B* and *SI Appendix*, Fig. S15 *A* and *B*). Moreover, in bilateral PoLN metastasis models with 4T1-Luc-TROP2 and 4T1-Luc-NC, TROP2-high PoMLNs exhibited a ~1.63-fold higher MFI than TROP2-low PoMLNs at 12 h postinjection of TTP-ICG (*P* < 0.01; Fig. 4 *C* and *D* and *SI Appendix*, Fig. S15*C*).

Following TTP-ICG subcutaneous injection in unilateral PoLN metastasis models (4T1-Luc-TROP2 or MDA-MB-231-Luc-TROP2), PoMLNs demonstrated significantly elevated MFI compared to nonmetastatic PoLNs (PoNLNs), with maximal differences evident at 4 h postinjection (4T1-Luc-TROP2: 65.40 ± 2.66 vs. 21.44 ± 11.48, *P* < 0.0001; MDA-MB-231-Luc-TROP2: 50.98 ± 4.11 vs. 24.75 ± 5.88, *P* < 0.0001; *SI Appendix*, Fig. S16).

To comprehensively validate the diagnostic performance of TTP-ICG for metastatic lymph nodes (MLNs) detection, a systematic evaluation involving 17 mice with established unilateral PoLN metastasis was conducted. At 4 h postinjection of TTP-ICG, PoMLN displayed elevated fluorescence compared to PoNLN, while the metastatic sacral lymph node (SaMLN), missed by bioluminescence (BL) imaging, also showed brighter fluorescence than nonmetastatic sacral lymph node (SaNLN; Fig. 4 *E* and *F*). As depicted in Fig. 4 *G* and *H*, all 17 PoLNs on the tumor-bearing side, confirmed as tumor metastasis, presented a ~2.82-fold higher MFI compared to contralateral PoNLNs (*P* < 0.0001) with an AUC of 0.952 (95% CI: 0.882 to 1.000). When setting the MFI threshold at 21, we observed 100% sensitivity, with nearly half of PoNLNs (47.06%, 8/17) falling below this cutoff. Moreover, for sacral lymph nodes (SaLNs), metastatic involvement (confirmed in 11/17 mice) showed a ~2.10-fold higher MFI than nonmetastatic counterparts (*P* < 0.0001; Fig. 4*I*) with an AUC of 0.953 (95% CI: 0.886 to 1.000; Fig. 4*J*).

To assess the early detection capability of TTP-ICG for metastatic lesions, mice with various modeling times (10, 15, 20, and 25 d) were subcutaneously injected with TTP-ICG in foot pads. Pathological examination revealed that early metastasis (0.7 mm in diameter) first appeared at 15 d postinoculation, which was successfully detected by TTP-ICG-based NIR-II fluorescence imaging (Fig. 4 *K* and *L*). With molding times of 15, 20, and 25 d, the tumor-bearing-side PoLNs, pathologically confirmed as MLNs, demonstrated elevated NIR-II fluorescence compared to contralateral controls. Intriguingly, NIR-II imaging identified a micrometastatic PoLN in mice with 15 d of modeling and a micrometastatic SaLN at 25 d of modeling which were undetected by BL imaging (*SI Appendix*, Fig. S17).

### TTP-ICG-Based RIIM Facilitated the Identification of Breast Tumor Tissue via Ex Vivo Imaging.

**Fig. 5.**
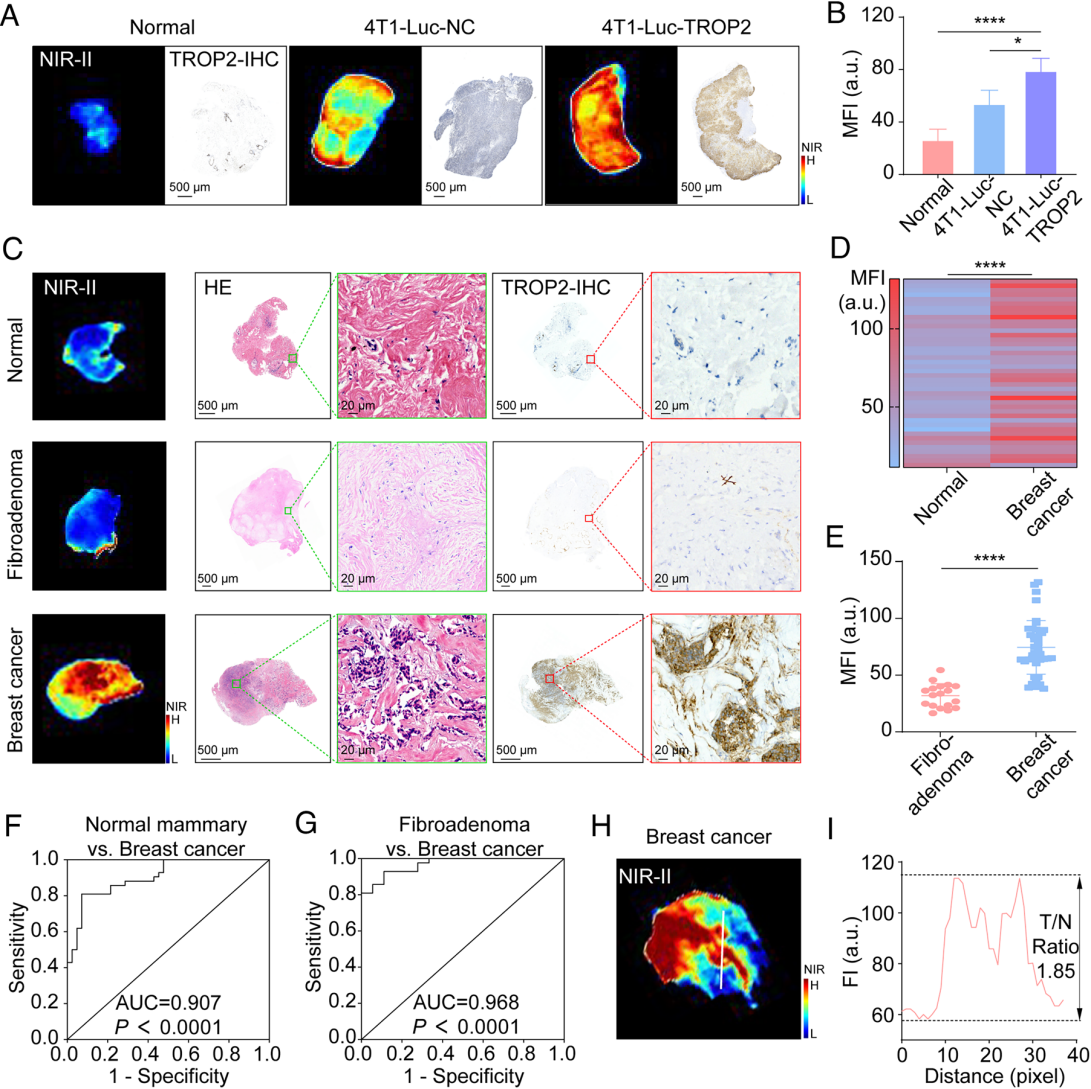
TTP-ICG-based RIIM to distinguish BC and normal mammary gland. (*A* and *B*) Representative ex vivo NIR-II images, TROP2-IHC (*A*), and MFI analysis (*B*) of normal mammary gland, 4T1-Luc-NC and 4T1-Luc-TROP2 tumors from mouse model after RIIM (n = 4). (*C*) Representative ex vivo NIR-II images and pathological analyses of normal mammary gland, fibroadenoma, and BC tissues from patients after RIIM. (*D*) MFI analysis of 42 pairs of normal mammary gland and BC tissues from patients after RIIM. (*E*) MFI analysis of fibroadenoma (n = 18) and BC (n = 42) tissues from patients after RIIM. (*F*) ROC curve of normal mammary gland and BC tissues according to the pathology results and MFI from (*D*). (*G*) ROC curve of fibroadenoma and BC tissues according to the pathology results and MFI from (*E*). (*H* and *I*) NIR-II image of a representative BC tissue (*H*) and the fluorescent intensity (*I*) across the LOI (the white line). (*P < 0.05, ****P < 0.0001, mean ± SD).

To expedite the clinical application of TTP-ICG, we have devised an ex vivo RIIM for intraoperative discrimination of BC. Initial optimization studies using 4T1-Luc-TROP2 tumor demonstrated that brief incubation (3 to 10 min) with TTP-ICG (5 to 20 μg/mL) yielded significantly higher MFI in tumor tissue compared to normal breast tissues (*SI Appendix*, Fig. S18). Notably, 20 μg/mL TTP-ICG incubation for just 3 min yielded an optimal TNR of 3.03 ± 0.62. Under this incubation condition, 4T1-Luc-TROP2 tumors (high-TROP2) also demonstrated significantly higher MFI than both 4T1-Luc-NC tumors (low-TROP2) and normal breast tissues (79.02 ± 9.63 vs. 53.88 ± 10.41 vs. 26.40 ± 8.17; *P* < 0.0001; Fig. 5 *A* and *B*).

Clinical validation in human specimens confirmed these findings, with the same optimal incubation parameters yielding a peak TNR of 1.91 ± 0.2 between malignant and normal breast tissues (*SI Appendix*, Fig. S19). Under this incubation condition, BC tissue exhibiting the highest TROP2 expression demonstrated superior MFI than normal mammary and fibroadenoma tissues (Fig. 5*C*). Comparative analysis of 42 matched normal-tumor tissue pairs revealed significantly elevated levels of both MFI (74.42 ± 23.59 vs. 39.88 ± 13.90; *P* < 0.0001) and H-Score (126.13 ± 53.78 vs. 47.74 ± 23.20; *P* < 0.0001) in BC tissues (Fig. 5*D* and *SI Appendix*, Fig. S20*A*). Furthermore, linear regression and correlation analysis demonstrated a strong positive correlation between the H-Score and MFI (r = 0.8920; R^2^ = 0.7956; *P* < 0.0001; *SI Appendix*, Fig. S20*B*). ROC analysis demonstrated excellent diagnostic performance (AUC = 0.907; 95% CI: 0.846 to 0.968; sensitivity = 81.0%, specificity = 92.9% at MFI cutoff = 60.12; Fig. 5*F* and *SI Appendix*, Table S2). The MFI threshold of 60.12 successfully identified all TROP2-high/moderate BC tissues (28/28, H-Score > 100) and 43% TROP2-low cases (6/14, H-Score < 100), and 50% of the missed cases (4/8) showed H-Score < 50. Besides, all specimens (18 of 84) above the MFI threshold of 78 were confirmed BC tissues with 100% specificity, while those (22 of 84) below the threshold of 38 were normal tissues with 100% sensitivity.

Further, the MFI obtained after RIIM closely aligned with endogenous TROP2 expression levels across various BC subclasses (*SI Appendix*, Fig. S21*A*). TNBC tissues exhibiting the highest TROP2 expression yielded the strongest MFI, whereas normal breast glands with the lowest TROP2 levels showed the weakest signal. The HR+/HER2− and HER2+ subtypes exhibited intermediate TROP2 expression and correspondingly moderate MFI. Analysis of TROP2 expression across subtypes (*SI Appendix*, Fig. S21*B*) revealed high level in TNBC (100% TROP2-high/moderate, H-Score > 100), intermediate level in HR+/HER2- BC (69.2% TROP2-high/moderate), and relatively low level in HER2+ BC (50% TROP2-high/moderate). Moreover, the trend in MFI across subtypes closely aligned with the TROP2 expression pattern (*SI Appendix*, Fig. S21*C*): Normal: 39.88 ± 13.90, HER2+: 57.58 ± 17.93, HR+/HER2-: 72.28 ± 18.04, and TNBC: 114.28 ± 19.74. Collectively, all BC cases in our cohort exhibited TROP2 expression and corresponding NIR-II fluorescence following RIIM. Importantly, clear NIR-II signals were observed even in cases with TROP2-low levels, such as those found in some HER2+ and HR+/HER2- cases.

Moreover, this RIIM distinctly differentiated BC from fibroadenomas, with BC tissues showing a ~2.34-fold higher MFI compared to 18 fibroadenoma samples (*P* < 0.0001; Fig. 5*E*). ROC analysis yielded an AUC of 0.968 (95% CI: 0.932 to 1.0; sensitivity = 92.9%, specificity = 88.9% at MFI cutoff = 41.65; Fig. 5*G*).

Spatial fluorescence mapping along tumor-normal interfaces revealed ~1.85-fold higher signal in cancerous regions vs. adjacent normal tissue (Fig. 5 *H* and *I* and *SI Appendix*, Fig. S22). Fluorescence intensity strongly correlated with histopathological features (H&E) and TROP2 expression patterns, validating the RIIM’s precision for intraoperative margin assessment.

### TTP-ICG-Based RIIM Facilitated the Identification of mSLNs via Ex Vivo Imaging.

**Fig. 6.**
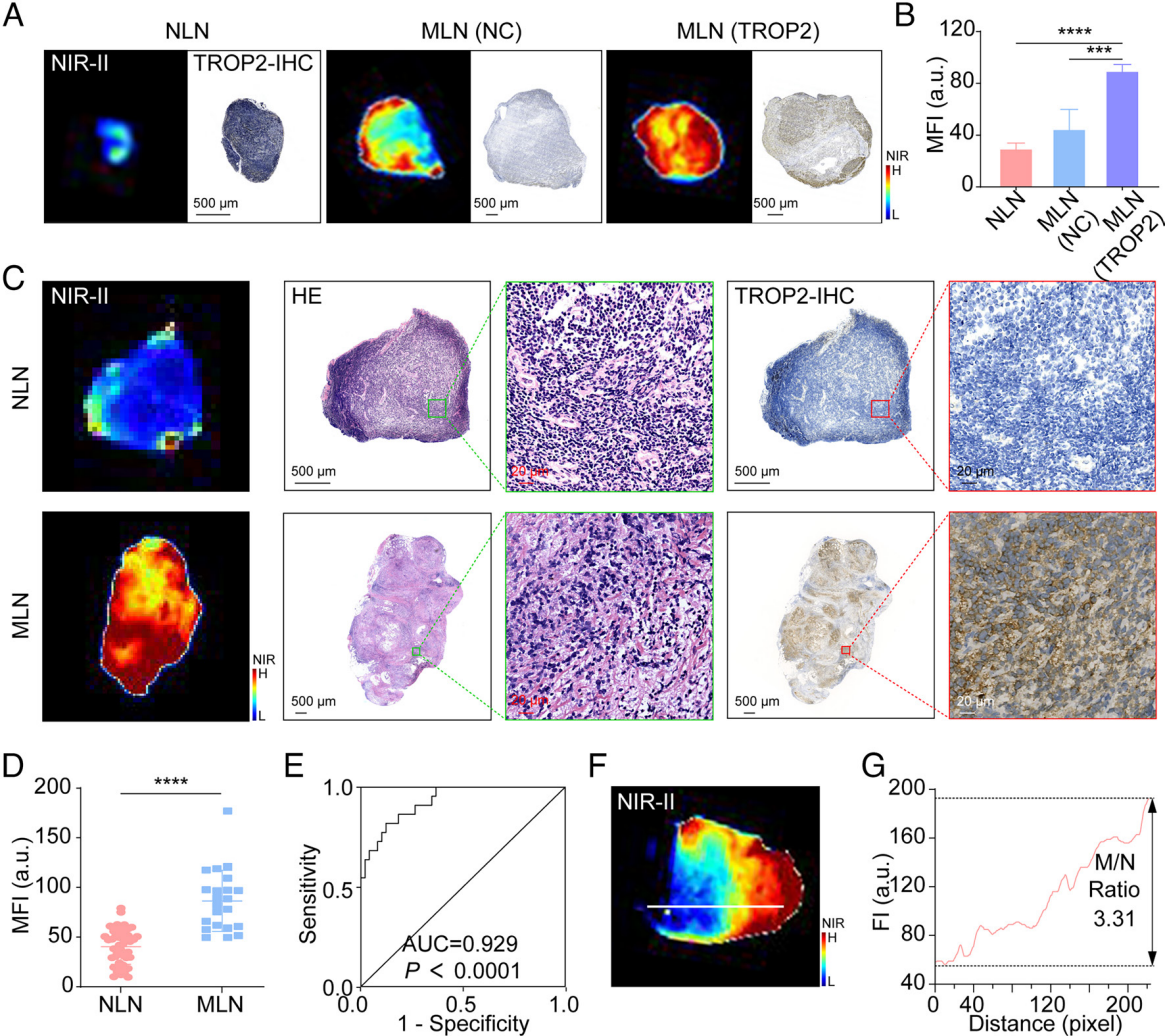
TTP-ICG-based RIIM to differentiate non- and metastatic SLNs. (*A* and *B*) Representative ex vivo NIR-II images, TROP2-IHC (*A*) and MFI analysis (*B*) of NLN, 4T1-Luc-NC MLN, and 4T1-Luc-TROP2 MLN tissues from mouse model after RIIM (n = 4). (*C*) Representative ex vivo NIR-II images and pathological analyses of non- and metastatic LN tissues from patients after RIIM. (*D*) The MFI analysis of NLNs (n = 49) and MLNs (n = 22) tissues from patients after RIIM. (*E*) ROC curve of NLNs and MLNs according to the pathology results and MFI from (*D*). (*F* and *G*) NIR-II image of a representative MLN tissue (*F*) and the fluorescent intensity (*G*) across the IOL (the white line). (*** *P* < 0.001, ****P < 0.0001, mean ± SD).

The feasibility of RIIM for differentiating mSLNs from nonmetastatic SLNs was first evaluated using tissues derived from 4T1-Luc-TROP2 PoLN metastasis models. The 4T1-Luc-TROP2 MLNs exhibited superior MFI than NLNs (*SI Appendix*, Fig. S23). Notably, when incubated with 20 μg/mL TTP-ICG for 3 min, the MLNs-to-NLNs (M/N) ratio peaked at ~3.22-fold. Moreover, MLNs with high TROP2 expression (4T1-Luc-TROP2) exhibited significantly higher MFI than low-TROP2 MLNs (4T1-Luc-NC) and NLNs (89.72 ± 4.82 vs. 44.77 ± 15.05 vs. 29.73 ± 4.14; *P* < 0.0001; Fig. 6 *A* and *B*).

71 pieces of LNs from BC patients were then gathered to perform RIIM. According to the pathologic analysis, 22 of 71 LNs specimens confirmed metastasis (MLNs) with elevated TROP2-expression, exhibiting higher MFI than those without metastasis (NLNs, 86.50 ± 30.75 vs. 40.29 ± 17.70; *P* < 0.0001; Fig. 6 *C* and *D*). Furthermore, the MFI and H-Score showed a strong positive correlation, as demonstrated by linear regression analysis (r = 0.8746; R^2^ = 0.7650; *P* < 0.0001; *SI Appendix*, Fig. S24). ROC analysis revealed strong diagnostic performance (AUC = 0.929; 95% CI: 0.871 to 0.988; sensitivity = 81.8%, specificity = 87.8% at MFI cutoff = 59.14; Fig. 6*E* and *SI Appendix*, Table S3). The MFI threshold of 59.14 successfully identified all TROP2-high/moderate MLN tissues (15/15, H-Score > 100) and 43% TROP2-low cases (3/7, H-Score < 100). Moreover, all specimens (12 of 71) above the MFI threshold of 80 were confirmed metastatic with 100% specificity, while those (31 of 71) below the threshold of 49 were nonmetastatic with 100% sensitivity.

Spatial analysis along the line of interest (LOI) demonstrated that metastatic foci with elevated TROP2 expression exhibited ~3.31-fold higher fluorescence than adjacent normal regions (Fig. 6 *F* and *G* and *SI Appendix*, Fig. S25). H&E staining confirmed the colocalization of NIR-II signals with metastatic lesions, underscoring RIIM’s precision for intraoperative identification of mLNs.

## Discussion

In the present study, we report the development of a NIR-II fluorescence probe TTP-ICG by conjugating ICG dye with the TROP2-targeting peptide TTP, which was identified through phage display screening of CPPC-paired disulfide-rich peptide libraries as described previously (43). Our results confirmed that TTP-ICG was specifically internalized by TROP2-expressing cells, and the binding/internalization was competitively inhibited by incubation with excess free TTP, demonstrating its high TROP2-targeting specificity and binding affinity. Moreover, this NIR-II probe allows us to intraoperatively evaluate surgical margins during BCS and visualize mSLNs when performing SLNB, thus enabling simultaneous margin assessment and mSLNs identification in breast cancer surgery.

In our previous study, we have generated cyclic-RGD peptides-based nano-probes for integrin αvβ3-targeted NIR-II fluorescence imaging (45–47), which could differentiate tumors from normal tissues (AUC of ROC: 0.890 to 0.978). Herein, TTP-ICG exhibited superior discrimination between tumor and normal tissues with higher TNR than that reported with RGD probes and showed a better diagnostic performance in MMTV-PyVT transgenic mice (AUC 0.983). Especially, TTP-ICG could effectively visualize tumors with moderate or heterogeneous TROP2 expression in the transgenic model. Remarkably, with the detection of 100% of small tumors ~2 mm in diameter (39/39), TTP-ICG guided complete tumor resection, reduced local recurrence, and improved survival outcomes in animal models. These findings keep consistent with our previous work using the ICG-conjugated Sacituzumab Govitecan (ICG-SG), an antibody-based probe, for TROP2-targeted NIR-II imaging, further supporting TROP2’s role as a suitable target for breast cancer imaging (36). However, as reported, antibody-based probes face inherent limitations, including poor tumor penetration and prolonged background signals owing to their large size (48, 49). In contrast, with its small molecular weight, TTP-ICG demonstrated excellent biocompatibility such as rapid metabolism and renal clearance.

In addition, as a protein expressed in epithelial cells (32), TROP2 could serve as an indicator of lymph node metastasis just like pan-CK proteins have done. In this study, we explored TTP-ICG’s capability to visualize the mSLNs with NIR-II fluorescence imaging both in vivo and ex vivo. Surprisingly, the NIR-II imaging probe achieved pretty high diagnostic accuracy (AUC > 0.95) in differentiating metastatic from nonmetastatic lymph nodes across multiple murine models of lymph node metastases, including both popliteal (PoLNs) and sacral lymph nodes (SaLNs). Interestingly, the technology exhibited high sensitivity in detecting micrometastases, defined as tumor deposits less than 1 mm in diameter. Most importantly, a set cutoff for MFI with a threshold of 21 yielded 100% sensitivity while correctly classifying 47% of negative nodes, suggesting this approach could potentially eliminate unnecessary SLNB procedures in nearly half of node-negative cases.

As described above, we have convincingly demonstrated TTP-ICG’s significance for real-time intraoperative navigation in breast cancer surgery, including accurate tumor margin delineation and mSLNs identification in animal studies. In order to facilitate intraoperative decision-making, we have developed an ex vivo incubation protocol RIIM for assessing surgical margins with ICG-SG (36). In the present multicenter investigation, we significantly optimized the method and shortened the RIIM procedure’s time to ~8 min from 15 min when using TTP peptide as a targeting element. This optimized approach is notably faster than both frozen section (average, 27 min) and imprint cytology (average, 13 min), while maintaining a comparable sensitivity and specificity (13).

The improvement in the intraoperative incubation method is certainly critical for timely decision-making, especially for determining the necessity of re-excision during BCS. Herein, we utilized RIIM with machine-based MFI thresholds, demonstrating a strong correlation with TROP2 expression. Our results further confirmed previous findings, i.e., TROP2 is highly expressed in approximately 90% of BC (50), with particularly frequent in TNBC (> 95%, 51–53) and relatively lower in HER2+ BC (54, 55). Considering the heterogeneity of TROP2 expression found in >50% of tumor cells (51), to improve the accuracy and reliability, we implemented a dual-threshold strategy, providing a clear diagnostic framework: tissues with MFI above 78 as malignant, those below 38 as normal. This approach could potentially eliminate the need for frozen-section analysis in nearly half of clear-cut cases, significantly improving surgical efficiency.

In addition, we have successfully applied the dual-threshold method to detect MLNs. Distinct thresholds were employed here due to various TROP2 expression across different tissue types (breast vs. LNs, primary tumor vs. metastasis) (56), resulting in 100% identification of MLNs when MFI > 80 and 100% of NLNs when MFI < 49. Thus, more than 60% of SLNs will be identified as metastasized or unmetastasized ones based on these criteria. Compared with frozen-section pathology or OSNA assays, this approach not only delivers rapid results but also preserves tissue integrity. Thus, our findings do very likely demonstrate the possibility of substituting frozen pathology with intraoperative NIR-II fluorescence imaging, and subsequently lead to decision-making of Go/No Go for further axillary procedure at certain circumstances.

Within the contemporary management paradigm for EBC, the sequential implementation of SLNB and BCS has gained widespread acceptance as an effective approach for precise surgical intervention (57). One important scenario is to apply the TTP-ICG-based RIIM to SLNB procedure, and then to evaluate surgical margins by bypassing intraoperative pathology in cases where tissues’ fluorescence meets predefined diagnostic thresholds during EBC surgery.

However, our investigation also has several limitations. First, the current study is limited by a relatively small sample size, requiring an expanding enrollment to refine the method and assess the re-excision rates compared with other procedures. Second, while RIIM offers a solution to the challenges posed by the clinical translation of systematic administration, further exploration of the feasibility of in vivo application requires additional advancements, including comprehensive safety assessments in large animal models. Third, preoperative TROP2 IHC may help to identify appropriate patients for imaging because our protocol carries a risk of false negatives. Moreover, the technology may prolong assessment time for indeterminate MFI cases and impact on subsequent frozen section. Further efforts should be made to shorten the RIIM time, increase its accuracy through AI-based analysis (58), and evaluate its impact on pathology.

Taken together, the present work establishes a promising bifunctional NIR-II fluorescence imaging platform for simultaneous assessment of both surgical margins and nodal involvement in EBC patients during BCS. As the BCS procedure plays an increasingly important role toward precision surgery, such innovative technology is strongly demanded in achieving optimized cosmetic appearances and better clinical outcomes. Future studies should focus on expanding clinical validation cohorts, developing multiplexed targeting strategies for TROP2-low tumors, and advancing toward in vivo administration paradigms.

## Materials and Methods

### Differentiation of Benign and Malignant Tissues in the Transgenic Murine Model.

MMTV-PyVT mice (n = 4) and wild-type mice (n = 4) were intravenously administered TTP-ICG (5 mg/kg) and killed at 48 h postinjection. NIR-II fluorescence imaging was then performed using a small-animal imaging system (DPM-IVFM-NIR-II, DPM, Zhuhai, CN) with 808 nm laser excitation (0.1 W/cm^2^), a 1,000 nm long-pass filter, and 150 ms exposure. Sequentially, five pairs of mammary glands were resected under NIR-II image guidance. All excised tissues were collected for ex vivo NIR-II imaging and subsequent histological analysis.

### Fluorescence-Guided Tumor Resection in the Intramuscular Tumor-Invasion Model.

Following BL imaging, mice bearing muscle-invasive tumors (n = 14) were intravenously administered with TTP-ICG and subjected to NIR-II fluorescence imaging at 48 h postinjection. Tumors were then resected under white-light guidance. Seven mice were randomly selected to assess residual NIR-II fluorescence signals in the tumor bed. Tissues exhibiting NIR-II fluorescence were carefully excised until no detectable signals remained. All resected tissues were collected for ex vivo NIR-II imaging and subsequent histological analysis. Postoperative intraperitoneal antibiotics were given to prevent infection. BL imaging was repeated 14 d after surgery to monitor recurrence, and survival was tracked for 45 d.

### Distinguishment of Non- and Metastatic SLNs in the Unilateral PoLN Metastasis Model.

Mice bearing unilateral 4T1-Luc-TROP2 or MDA-MB-231-Luc-TROP2 PoLN metastasis (n = 4) were subcutaneously injected with TTP-ICG into bilateral hind foot pads. NIR-II fluorescence imaging was conducted at 1, 2, 4, 8, 12 h postinjection to explore the optimal imaging time point.

Unilateral 4T1-Luc-TROP2 PoLN metastasis mice (n = 17) were subcutaneously injected with TTP-ICG (0.25 mg/kg) into bilateral hind foot pads. NIR-II fluorescence imaging was performed 4 h postinjection. The PoLNs and SaLNs were then collected for histological analysis to distinguish metastasis status.

At 10, 15, 20, and 25 d after 4T1-Luc-TROP2 inoculation into the unilateral hind foot pad, mice were subcutaneously injected with TTP-ICG (0.25 mg/kg) and imaged 4 h after injection.

### Patient Enrollment.

This study involved 48 BC (*SI Appendix*, Table S1) and 11 fibroadenoma patients from Yunnan Cancer Hospital and the Affiliated Cancer Hospital of Shantou University Medical College. The study protocol was approved by the Institutional Ethical Review Board and registered on ClinicalTrials.gov (NCT06713681). Written informed consent was obtained from all participants prior to their enrollment in the study.

### TTP-ICG-Based RIIM in Human Tissues.

Fresh BC, normal breast gland, fibroadenoma, and LN tissues were obtained during surgery. Fresh BC and normal breast gland tissues (n = 4) were first immersed in the TTP-ICG solution at varying concentrations (5, 10, 20 μg/mL) and immediately agitated at room temperature for 3, 5, or 10 min. After 5 min of rinsing, NIR-II imaging was performed to explore the optimum incubation condition. Subsequently, BC, normal breast gland, fibroadenoma, and LN tissues were incubated using the identified optimal protocol. The dual thresholds were set through the following criteria: The high threshold was defined as the maximum MFI observed in normal breast/LN tissues, while the low threshold was set as the minimum MFI from tumor/metastatic tissues. These fresh tissues received RIIM were then fixed and sectioned after NIR-II imaging. The histology results were derived from the subsequently prepared slides of these tissues.

## Supplementary Material

Appendix 01 (PDF)

Dataset S01 (XLSX)

## Data Availability

Study data are included in the article and/or supporting information.
