# Supplementary material for: TROP2-targeted NIR-II fluorescence imaging for visualizing surgical margins and metastatic sentinel lymph nodes in breast cancers
Source: Proc Natl Acad Sci U S A. 2026 Feb 19;123(8):e2519420123. doi: 10.1073/pnas.2519420123 (PMC12933058; doi:10.1073/pnas.2519420123)
Supplement: Supplementary file 1 — Appendix 01 (PDF) [file pnas.2519420123.sapp.pdf]

Supporting Information for

TROP2-targeted NIR-II fluorescence imaging for visualizing surgical margins and metastatic sentinel lymph nodes in breast cancers

Kang-Liang Lou, Jing-Wen Bai, Hong-Tan Liu, Lin-Ling Lin, Cheng-Xi Li, Yi-Yang Gao, Sheng-Jie Lin, Yi-Fei Pei, Xiao-Long Wei, Yi-Xin Chen, Yi-Yin Tang, Hong-Yu Chen, Zhi-Yao Li, Rong Guo, Shi-Cong Tang, Chuan-Liu Wu, Guo-Jun Zhang

Corresponding authors: Guo-Jun Zhang and Chuan-Liu Wu

E-mail: zhangguojun@kmmu.edu.cn and chlwu@xmu.edu.cn.

This PDF file includes:

Supporting text
Figures S1 to S25
Tables S1 to S3
SI References

Supporting Information Text

Supplementary Materials and Methods

Synthesis and characterization of TTP-ICG

ICG-NHS (10 μmol, J&K Scientific, Beijing, CN) and TTP (8 μmol) were dissolved in 2 mL DMF. The pH of the mixture was adjusted to 8.0 with N,N-Diisopropylethylamine (DIEA, Aladdin, Shanghai, CN). After stirring at room temperature for 4 h, the product TTP-ICG was purified by high-performance liquid chromatography (HPLC, LC20-AD, Shimadzu, Kyoto, JPN). After lyophilization, the product was further analyzed by HPLC and LC-MS instrument (Autoflex max MALDI-TOF MS, Bruker, Karlsruhe, GER). Subsequently, the absorption and emission spectra were detected by UV-Vis-NIR spectrophotometry (Agilent, Beijing, CN), fluorescence spectrophotometer (Cary Eclipse, Agilent, Beijing, CN) and FLS980 Spectrometer (Edinburgh, Livingston, UK). The sequence of TTP is GSGSGCPPCGRWLECYDFNECELIDWCPPC-NH2. Besides, a nonsense peptide was also labeled with ICG to serve as contrast probe (CP-ICG).

Cell culture

MCF-7 and MCF-10A cell lines were purchased from the American Type Culture Collection (ATCC Manassas, VA, USA), 4T1-Luc and MDA-MB-231-Luc cell lines were purchased from the Zhong Qiao Xin Zhou Biotechnology Co., Ltd., Shanghai, CN. MCF-7 and MDA-MB-231-Luc cells were cultured in DMEM medium containing 10% FBS. 4T1-Luc cells were cultured in RPMI-1640 medium containing 10% FBS. MCF-10A cells were cultured in MCF 10A cell medium (Procell, Wuhan, CN). All cells were cultured in a standard humidified cell culture incubator (37 °C, 5% CO_2).

The expression of TROP2: Western blotting

According to the manufacturer's guidelines, total proteins were extracted from cells using RIPA buffer (Sigma Aldrich, St. Louis, US) and a protease inhibitor cocktail (Roche, Shanghai, CN). A total of 30 µg of protein was loaded onto a 10% SDS-PAGE (Beyotime, Shanghai, CN). Electrophoresis was carried out at 120 V for one hour. Then, proteins were transferred to the PVDF membranes at 250 mA for the next two hours. Primary antibodies were incubated overnight at 4 °C on membranes after blocking with 5% non-fat milk for 30 min. Azure C300 (Azure Biosystems, Beijing, CN) was used image the Western blots. The primary antibody for TROP2 (ab214488) was purchased from Abcam, Cambridge, UK, and the primary antibody for β-actin (TA-09) was purchased from Beijing Zhongshan Golden Bridge Biotechnology Co., Ltd.

The expression of TROP2: Immunohistochemical (IHC) Staining and Evaluation

IHC staining was performed on 5-μm tissue sections according to the manufacturer's instructions. A rabbit monoclonal anti-TROP2 antibody (ab214488, Abcam) was incubated at a dilution of 1:1000.

Expression was semi-quantitatively assessed using the H-score method, which incorporates both staining intensity and the percentage of positive tumor cells. Staining intensity was graded as 0 (negative), 1+ (weak), 2+ (moderate), or 3+ (strong). The H-score was calculated by multiplying the percentage of cells stained at each intensity level by the corresponding intensity value and summing the results, yielding a range of 0 to 300. Based on the H-score, TROP2 expression was categorized as follows: TROP2-Low (0–100), TROP2-Moderate (100–200), and TROP2-High (200–300).

Cytotoxicity assay

MDA-MB-231-Luc, MCF-7, and MCF-10A cells were inoculated into 96-well plates with 3000 cells per well. After 24 h culturing, TTP-ICG according to concentration gradients (0, 50, 100, 200, 400, 800 nM) was added for 48 h incubation. According to the manufacturer's guidelines, Cell Counting Kit-8 solution (TargetMol, Massachusetts, US) was added and incubated for 2 h. The absorbance of the samples at 450 nm was measured with Multiskan SkyHigh microplate spectrophotometer (Thermo Fisher Scientific, Waltham, US) and the cell survival rate was calculated. Viability (%) = (A_t - A_b) / (A_0 - A_b) × 100% (A_t: absorbance value of treatment well; A_0: absorbance value of control well; A_b: absorbance value of blank well).

Hemolytic Test

The blood collected from BALB/c mice with EDTA-Na_2 (5 mM, Beyotime, Shanghai, CN) was washed with PBS and centrifuged (1500 rpm, 15 min at 4ºC) to remove excess white blood cells. Subsequently, 200 μL red blood cells (RBCs) was taken and 4.8 mL of PBS was added to obtain the 4% RBC. TTP-ICG according to concentration gradients (0, 50, 100, 200, 400, 800 nM) was added and another group with 0.1% Triton x-100 as positive control. The mixed systems were shaken at room temperature for 3 h, and then were centrifuged at 10000 rpm for 3min. Afterwards, 100 uL supernatant was taken from each group into 96-well plate and the absorbance of the samples at 570 nm was measured with a full-wavelength enzyme marker. Hemolysis rate was calculated following the formula Hemolysis rate (%) = (A_t - A_b) / (A_P - A_b) × 100% (A_t: absorbance value of treatment well; A_p: absorbance value of positive control; A_b: absorbance value of blank well). A hemolysis rate exceeding 5% was defined as hemolysis.

Cell transfection

To establish stable TROP2 knockdown cells (MCF-7-shTROP2), MCF-7 cells were transfected with the LV3 (H1/GFP & Puro)-shTACSTD2 (shTACSTD2: 5'-GGGGAAGTACAAGAAGGTGGA-3') lentivirus (Genepharma, Shanghai, China). The control cell line (MCF-7-shNC) was also developed with a vector containing a scramble sequence. 5×10^4 cells were inoculated into 24-well plates for 24 h and then incubated with 10 μL lentivirus supernatants as well as 5 μg/mL polybrene for another 24 h, followed by selection using puromycin at a concentration of 2 μg/mL. To acquire stable TROP2-overexpressed cell lines, 4T1-Luc-TROP2 and MDA-MB-231-Luc-TROP2 were developed by transfection of the pCMV-mCherry-TACSTD2(human)-Hyg plasmid, which was obtained from MiaoLing Plasmid Platform (Wuhan, CN), their control cell lines with Cherry (4T1-Luc-NC and MDA-MB-231-Luc-NC) or GFP (MDA-MB-231-Luc-GFP) were established with a control

plasmid. 5×10^4 cells were inoculated into 24-well plates for 24 h and then incubated with 5 μg/mL plasmid as well as 2.5 μL Lipo-2000 (Thermo Fisher Scientific, Waltham, US) for another 24 h, followed by selection using hygromycin at a concentration of 30 μg/mL for 4T1-Luc and 120 μg/mL for MDA-MB-231-Luc. The expression of TROP2 was then verified by western blotting.

Tumor targeting of TTP-ICG *in vitro*

To quantitatively compare the uptake of TTP-ICG and CP-ICG in MDA-MB-231-Luc-TROP2 cells, flow cytometry (FCM) was employed. MDA-MB-231-Luc-TROP2 cells were seeded in a 12-well plate at a density of 1×10^5 cells per well and cultured for 24 h. The medium was then replaced with fresh medium containing either TTP-ICG or CP-ICG at a concentration of 200 nM. After incubation for various time points (0, 1, 2, 4, 8, and 12 h), the cells were harvested, washed, and resuspended for FCM analysis (CytoFlexS, Beckman Coulter, US).

Subsequently, the difference in uptake for TTP-ICG in MCF-7-shNC, MCF-7-shTROP2 and the 1:1 mix of MCF-7-shNC and MCF-7-shTROP2 were analyzed via FCM and Odyssey® CLX two-color infrared laser imaging system (LI-COR, Nebraska, US). Cells were seeded in a 12-well plate (1×10^5 cells per well) and cultured for 24 h. The medium was then replaced with fresh medium containing TTP-ICG (200 nM). After 8 h of incubation, the plate was scanned using the Odyssey® CLX system. For FCM analysis, cells were collected after 8 h of incubation with TTP-ICG. Similar experiments were conducted with MDA-MB-231-Luc-TROP2, MDA-MB-231-Luc-NC, and their mixed system, as well as with 4T1-Luc-TROP2, 4T1-Luc-NC, and their mixed system. To analyze the differences in cell uptake more intuitively, 4T1-Luc-NC, 4T1-Luc-TROP2, MCF-7-shNC and MCF-7-shTROP2 cells were respectively seeded to a 12-well chamber slide (Jing-An Biological, Shang Hai, CN, 1×10^5 cells per well) and cultured for 24h. Afterward, the cells were all incubated with TTP-ICG for 8 h. After being washed with PBS and fixed with 4% paraformaldehyde, cells were stained with Antifade Mounting Medium with DAPI (Beyotime, Shang Hai, CN). The fluorescence images of cells were acquired by the ortho fluorescence microscope (DM2700 P, Leica, Heerbrugg, Switzerland). Another blocking experiment was performed by incubating 4T1-Luc-TROP2 and MDA-MB-231-Luc-TROP2 cells with either TTP-ICG (200 nM) or TTP-ICG (200 nM) coupled with excess TTP (8 μM) for 8 h. After collection of cells, FCM was adopted to assess mean fluorescence intensity (MFI) of these cells.

In addition, MDA-MB-231-Luc-GFP (low TROP2-expression), MDA-MB-231-Luc-TROP2 (high TROP2-expression) and the balanced mix system of both cell lines were incubated with TTP-ICG (200 nM) for 8 h, followed by FCM analysis. Meanwhile, the balanced mix system was further incubated with equal ICG, CP-ICG and TTP-ICG (200 nM) respectively. After 8 h uptake, FCM and ortho fluorescence microscope were used to detect fluorescence in each group.

Animal preparation

All mice used in the present study were female. BALB/c mice and BALB/c nude mice were procured from Jiangsu Gempharmatech Co., Ltd, China. A pair of FVB/N-Tg(MMTV-PyVT)634Mul/J mice (PyVT) were purchased from Jackson Laboratory (Bar Harbor, ME) and

bred at the Laboratory Animal Center of Xiamen University. All animal experiments were conducted in compliance with the ethical guidelines and were approved by the Institutional Animal Care and Use Committee of Xiamen University. The study strictly adhered to the regulations and standards set forth by the Xiamen University Animal Study Committee for the humane care and use of laboratory animals.

Subcutaneous tumor model

BALB/c mice (female, aged 6–8 weeks) were injected subcutaneously with 4T1-Luc-TROP2 cells (1×10^6 /mouse) into the right side of the dorsal, BALB/c nude mice (female, aged 6–8 weeks) were injected with MDA-MB-231-Luc-TROP2 cells (1×10^7 /mouse) into the same place. When the volume of the tumor reached 200 mm^3 (volume of tumor = length × $width^2$ × 0.52), the mice underwent bioluminescence imaging (IVIS Lumina III, PerkinElmer, Massachusetts, US) and fluorescence imaging (DPM-IVFM-NIR-II, DPM, Zhuhai, CN).

Bilateral tumor model with different TROP2 expression

BALB/c mice (female, aged 6-8 weeks) were injected subcutaneously with 4T1-Luc-TROP2 cells (1×10^6 /mouse) and 4T1-Luc-NC cells (1×10^6 /mouse) into the bilateral sides of the dorsal respectively. BALB/c nude mice (female, aged 6-8 weeks) were injected subcutaneously with 4T1-Luc-TROP2 cells (1×10^7 /mouse) and 4T1-Luc-NC cells (1×10^7 /mouse) into the same place. When the volume of the tumor reached 200 mm^3, the mice were received bioluminescence imaging and fluorescence imaging.

Multiple microtumors model

4T1-Luc-TROP2 cells (1×10^5) were randomly injected into the multiple subcutaneous locations of the back of BALB/c mice (female, aged 6-8 weeks). 3 days after injection, mice were received bioluminescence imaging and fluorescence imaging.

Intramuscular tumor-invasion model

4T1-Luc-TROP2 cells (1×10^6 /mouse) were injected into the superficial muscle of the right hind leg of BALB/c mice (female, aged 6-8 weeks). MDA-MB-231-Luc-TROP2 (1×10^7 /mouse) were injected into the same position of BALB/c nude mice (female, aged 6-8 weeks). For MCF-7-Luc tumor model establishment, BALB/c nude mice (female, aged 6-8 weeks) were first subcutaneously implanted with release 17β-estradiol pellets (Innovative Research of America) in the left side of the neck. 5×10^6 MCF-7 cells were injected with Matrigel (BioCoat) into the same position of BALB/c nude mice (female, aged 6-8 weeks). When the volume of the tumor reached 300-400 mm^3, the mice were received tumor resection.

Transgenic mouse model of breast cancer

The MMTV-PyVT transgenic mouse model is characterized by the spontaneous development of breast cancer. These mice were bred by crossing positive transgenic mice with wild-type FVB/N mice in a specific-pathogen-free (SPF) environment. Genotyping was used to select transgenic positive mice according to our study published before *(1)*.

Popliteal lymph node (PoLN) metastasis model

4T1-Luc-TROP2 cells (1×10^6 /mouse) were injected into the right/bilateral hind foot pads of BALB/c mice (female, aged 6-8 weeks). MDA-MB-231-Luc-TROP2 cells (5×10^6 /mouse) were injected into the same position of BALB/c nude mice (female, aged 6-8 weeks). Bioluminescence imaging was performed to monitor metastatic status of popliteal lymph nodes.

Bilateral PoLN metastasis model with different TROP2-expression

4T1-Luc-NC (1×10^6 /mouse) and 4T1-Luc-TROP2 cells (1×10^6/mouse) were respectively injected into the left and right hind foot pad of BALB/c mice (female, aged 6-8 weeks). Bioluminescence imaging was performed to monitor metastatic status of PoLNs.

***In vivo* evaluation of TTP-ICG targeting**

Mice bearing unilateral 4T1-Luc-TROP2 or MDA-MB-231-Luc-TROP2 subcutaneous tumors were randomized into two groups (n = 4) and intravenously administered either TTP-ICG (5 mg/kg) or CP-ICG (equivalent ICG dose). NIR-II fluorescence imaging was performed at 1, 12, 24, 36, 48, 72, 96 hours post-injection using a small-animal imaging system (DPM-IVFM-NIR-II, DPM, Zhuhai, CN) with 808 nm laser excitation (0.1 W/cm^2), a 1000 nm long-pass filter, and 150 ms exposure.

For bilateral tumor models (n = 4) with different TROP2 expression subcutaneous tumors, TTP-ICG was administered intravenously and imaged at 48 hours post-injection.

Biosafety of TTP-ICG *in vivo*

BALB/c mice (female, aged 6-8 weeks) were randomly divided into four groups (PBS, 1 d, 7 d, 28 d; n = 4 for each group). Mice in 1 d, 7 d and 28 d group were intravenously administered with TTP-ICG (5 mg/kg), and blood samples were collected on day 1, day 7 and day 28 post-injection for blood routine and blood biochemical tests. Mice in PBS group received equal volumes of PBS and blood was collected on day 28 post-injection for the same tests. After blood collection, mice were euthanized and major organs (heart, liver, spleen, lung, kidney and brain) were collected for histological analysis.

BALB/c mice (female, aged 6-8 weeks) were randomly divided into four groups (PBS, 1 d, 7 d, 28 d; n = 4 for each group). Mice in 1 d, 7 d and 28 d group were subcutaneously injected with TTP-ICG (0.25 mg/kg) into the bilateral hind foot pads. The skin tissue from foot pad were collected at the corresponding time points and Masson staining was then performed. Mice in PBS group were received equal volumes of PBS and the skin was collected on day 28 post-injection for the same staining.

The biodistribution and pharmacokinetics of TTP-ICG

Mice bearing 4T1-Luc-TROP2 subcutaneous tumor (n = 4) were intravenously treated with TTP-ICG (5 mg/kg) and were euthanized at 48 h post-injection. Subsequently, major organs

and tissues (heart, liver, spleen, lung, kidney, brain, gut, muscle, skin, tumor) were collected and imaged under DPM-IVFM-NIR-II.

For the pharmacokinetic study, BALB/c mice (n = 4) were intravenously administrated with TTP-ICG (5 mg/kg). At each time point post-injection (1, 5, 20, 30, 45, 60, 90, 120, 240, 360, 480, 720 min), 50 μL blood samples were collected with EDTA anticoagulant, followed by centrifugation at 3000 rpm for 5 min to obtain the plasma. The MFI of the plasma was then measured under Odyssey® CLX two-color infrared laser imaging system to obtain decay curve.

Identification of multiple microtumors *in vivo*

After BL imaging, mice bearing multiple microtumors (n = 5) were intravenously administered with TTP-ICG and euthanized at 48 hours post-injection. NIR-II fluorescence images were then obtained to compare with the BL results. Guided by the NIR-II images, tumors were sequentially excised and collected for *ex vivo* NIR-II imaging and subsequent histological analysis.

Discrimination of mSLNs in the bilateral PoLN metastasis model

Mice with bilateral 4T1-Luc-TROP2 or MDA-MB-231-Luc-TROP2 PoLN metastases (n = 4) were subcutaneously injected with either TTP-ICG (0.25 mg/kg) or CP-ICG (equivalent ICG dose) into their hind foot pads. NIR-II imaging was conducted at 1, 2, 4, 8, 12 hours post-injection with an exposure time of 300 ms.

In a bilateral PoLN metastasis model (n = 4) with 4T1-Luc-NC or 4T1-Luc-TROP2 for PoLN metastasis, TTP-ICG was injected into both hind footpads, and NIR-II fluorescence imaging was performed four hours post-injection.

TTP-ICG-based rapid incubation imaging method (RIIM) in mice tissues

4T1-Luc-TROP2 tumors and normal breast glands or normal LNs and 4T1-Luc-TROP2 metastatic LNs were obtained from mouse models. Tissues (n = 4) were immersed in the TTP-ICG solution with different concentration (5, 10, 20 μg/mL) and were immediately shaken at room temperature for 3, 5 or 10 min. After incubation, tissues were transferred into PBST and shaken for 5 min. The NIR-II fluorescence images of tissues were then acquired with different exposure time (100, 150, 200, 300, 400, 500 ms).

Statistical analysis

Data were obtained from at least three independent measurements (n ≥ 3). Statistical analyses were performed using GraphPad Prism (Version 8.0.1, GraphPad Software Inc., La Jolla, CA). Fluorescence intensities for NIR-II imaging were quantified by SBR (DPM, Zhuhai, CN), Image Studio (LI-COR, Nebraska, US), and PSLViewer. Flow cytometry (FCM) data were analyzed with FlowJo (Version 10, BD, New Jersey, US). ROC curves were generated using IBM SPSS Statistics (Version 22.0, IBM, New York, US). Fluorescence intensities for tissue sections were measured with Image Studio (LI-COR, Nebraska, US). Statistical significance was assessed using Student’s t-test (or the Mann-Whitney U test for non-

parametric data), one-way ANOVA, two-way ANOVA, or linear regression and correlation analysis, with a significance threshold of $P < 0.05$. Data were presented as the mean ± SD (standard deviation). Statistical significance was represented as * $P < 0.05$, ** $P < 0.01$, *** $P < 0.001$, and **** $P < 0.0001$.

Figures

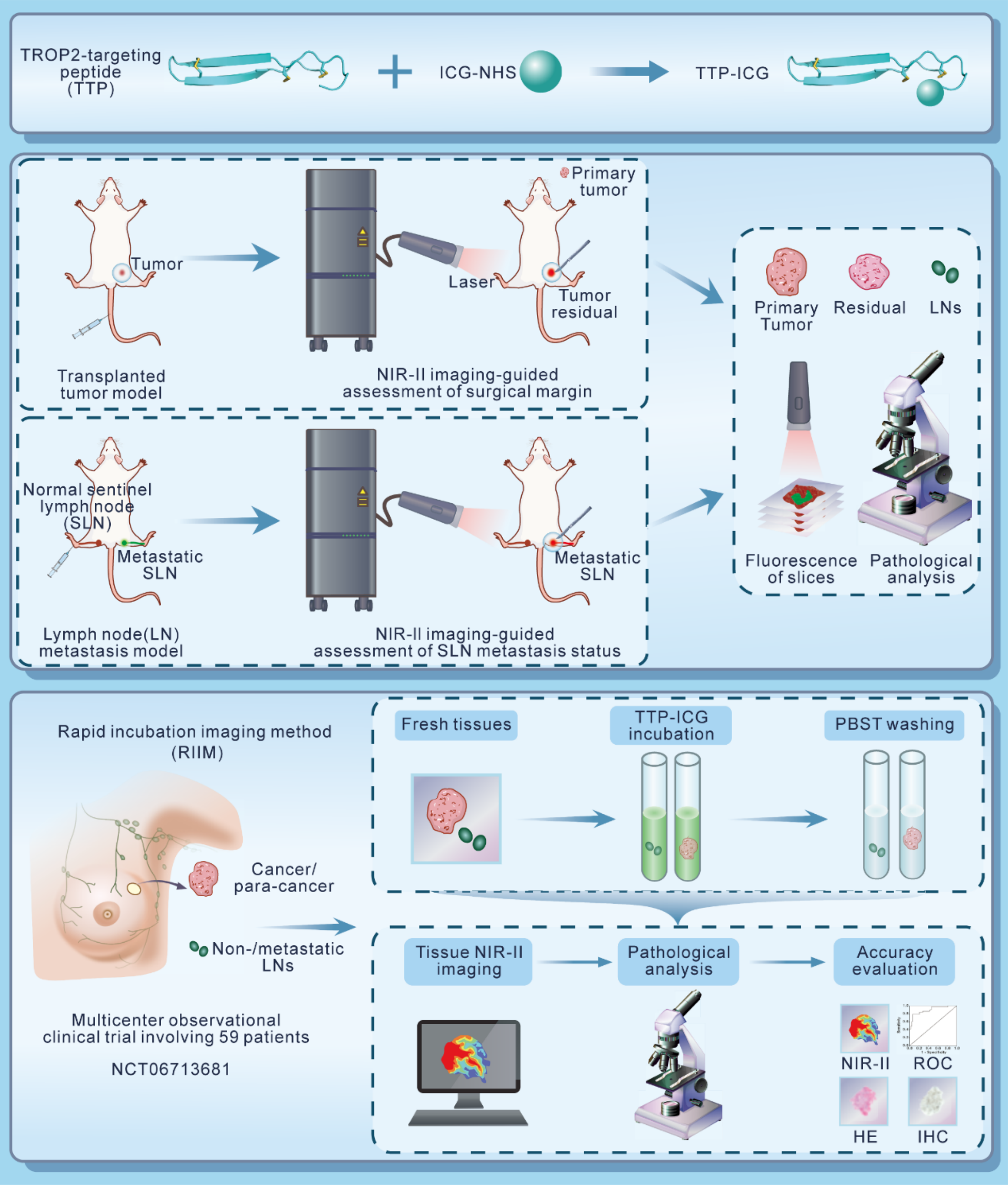

Fig. S1. Schematic illustration of TTP-ICG fabrication for NIR-II imaging-guided precise breast cancer (BC) surgery. Top panel depicts the synthesis process of TTP-ICG. Middle panel demonstrates in vivo NIR-II imaging-guided evaluation of surgical margin and sentinel lymph node (SLNs) metastasis status in mouse models. Bottom panel displays the clinical application of TTP-ICG-based rapid incubation imaging method (RIIM) for assessment of surgical margin and LNs metastasis status.

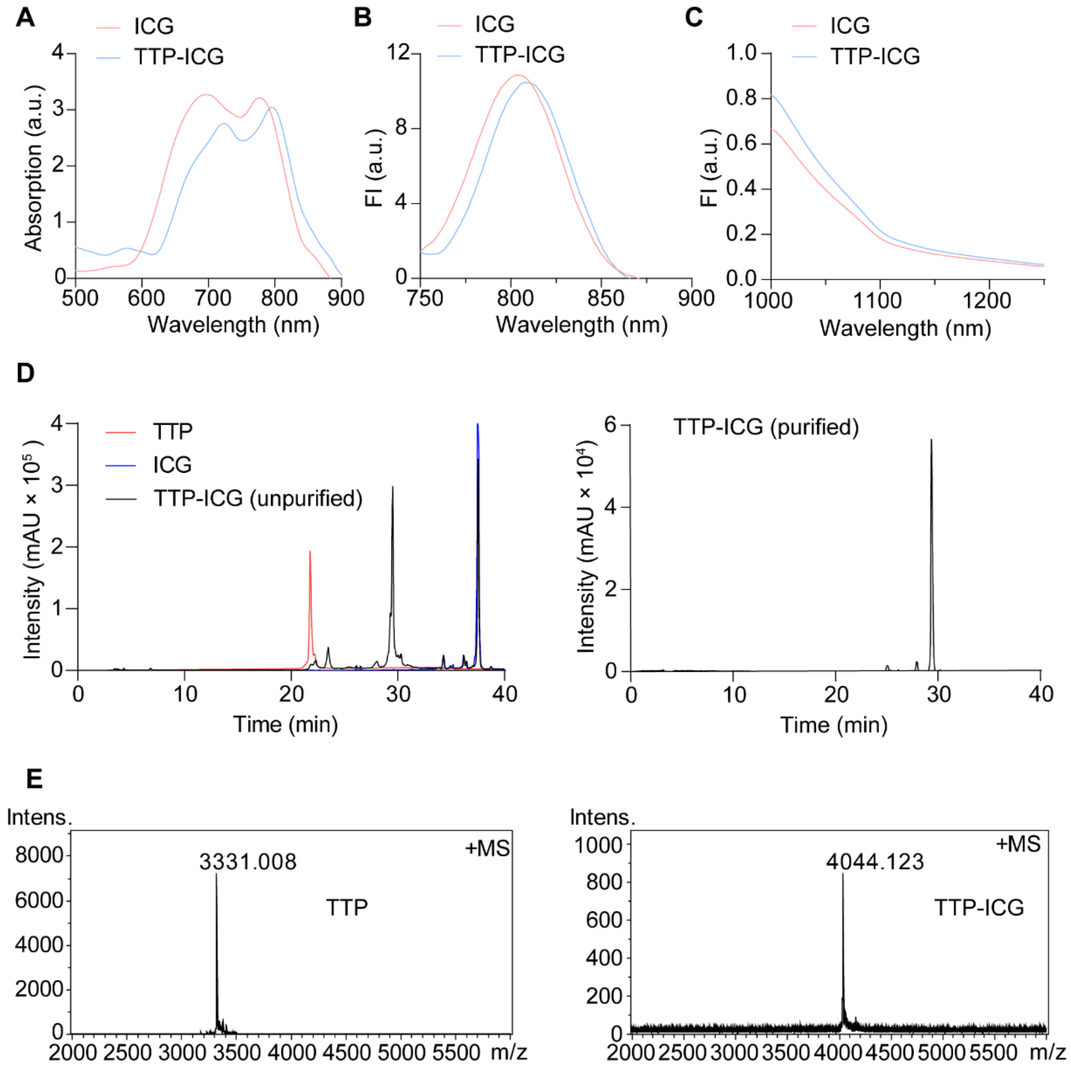

Fig. S2. Synthesis and characterization of TTP-ICG. (*A*-*C*) Absorption spectra (*A*), NIR-I emission spectra (*B*), and NIR-II emission spectra (*C*) of ICG and TTP-ICG. (*D*) High performance liquid chromatography (HPLC) results of TTP, ICG-NHS, unpurified TTP-ICG and purified TTP-ICG. (*E*) Mass spectrometry results of TTP and purified TTP-ICG.

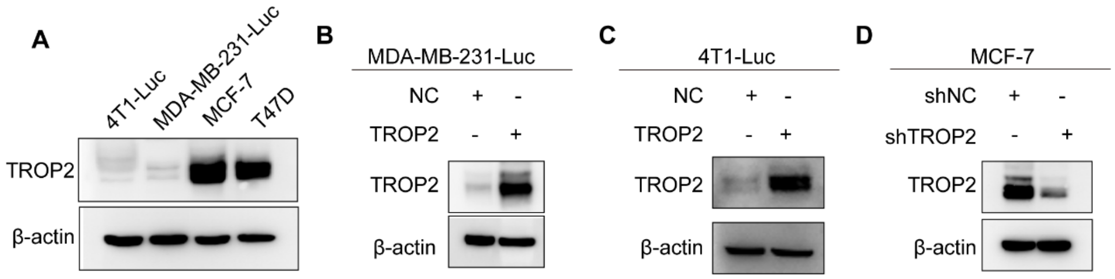

Fig. S3. TROP2 expression of different cell lines. (*A*) Western blotting (WB) validation of TROP2 expression in different breast cancer cell lines. (*B-D*) WB validation of TROP2 overexpression in MDA-MB-231-Luc cells (*B*), 4T1-Luc cells (*C*) and TROP2 knockdown in MCF-7 cells (*D*).

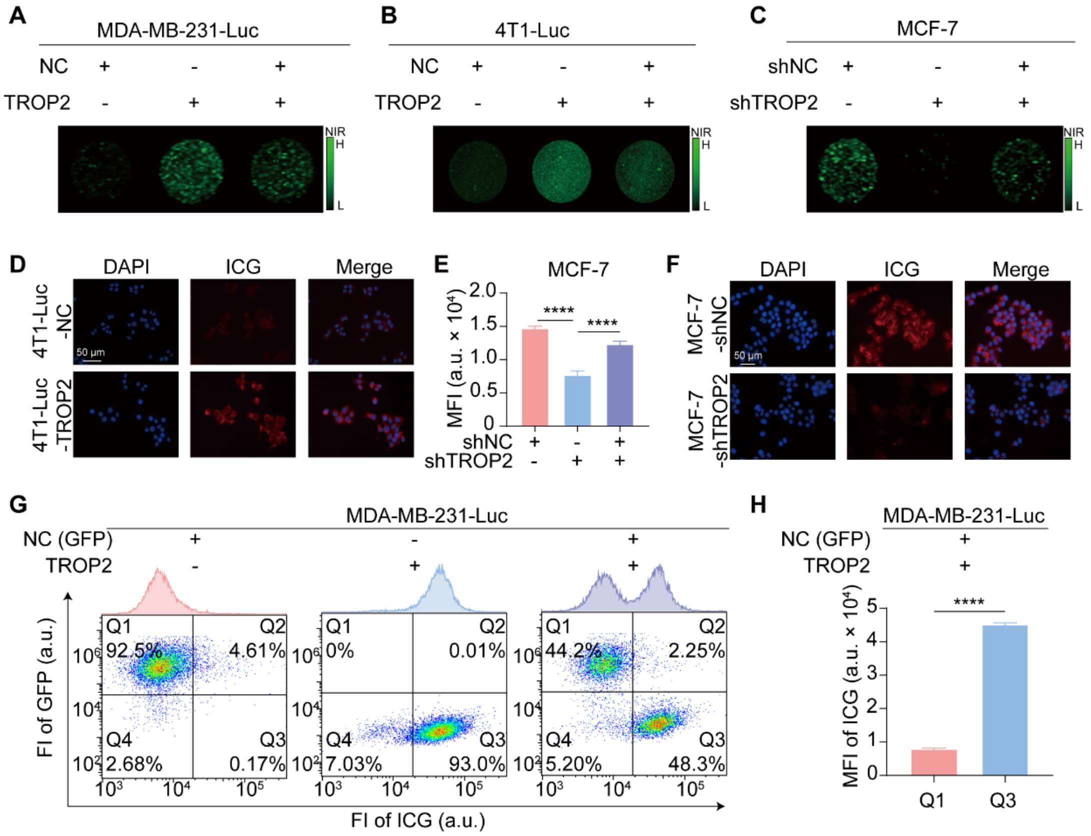

Fig. S4. TROP2-targeting validation of TTP-ICG *in vitro*. (*A*) Odyssey® fluorescence scanning images after incubating TTP-ICG with MDA-MB-231-Luc-NC cells, MDA-MB-231-Luc-TROP2 cells, and a 1:1 mixture of both cell lines. (*B*) Odyssey® fluorescence scanning images after incubating TTP-ICG with 4T1-Luc-NC cells, 4T1-Luc-TROP2 cells, and a 1:1 mixture of both cell lines. (*C*) Odyssey® fluorescence scanning images after incubating TTP-ICG with MCF-7-shNC cells, MCF-7-shTROP2 cells, and a 1:1 mixture of both cell lines. (*D*) Representative fluorescence microscopy images of 4T1-Luc-NC cells and 4T1-Luc-TROP2 cells treated with TTP-ICG. (*E*) Quantitative flow cytometry (FCM) analysis showing the mean fluorescence intensity (MFI) of MCF-7-shNC, MCF-7-shTROP2, and their 1:1 mixture after TTP-ICG treatment. (*F*) Representative fluorescence microscopy images after incubating TTP-ICG with MCF-7-shNC cells and MCF-7-shTROP2 cells. (*G*) FCM analysis after incubating TTP-ICG with MDA-MB-231-Luc-GFP cells, MDA-MB-231-Luc-TROP2 cells, or a 1:1 mixture of both cell lines. (*H*) Comparison of MFI in Q1 and Q3 populations in the mixed system incubated with TTP-ICG. (**** $P < 0.0001$, mean ± SD, n = 3)

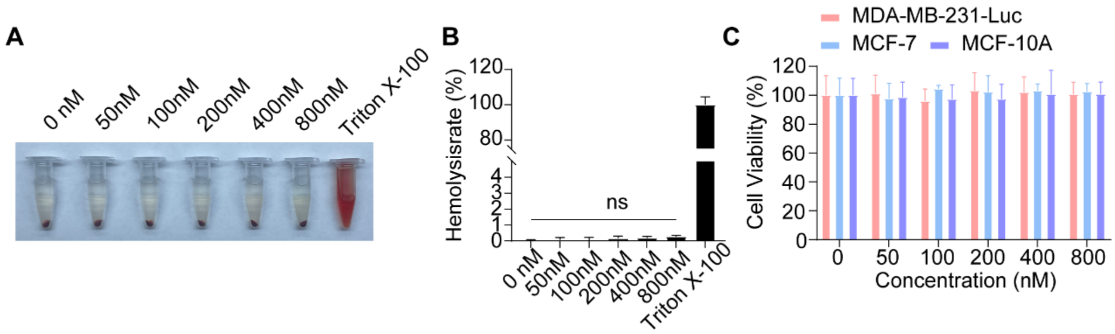

Fig. S5. Cytotoxicity of TTP-ICG. (*A* and *B*) Hemolysis assay of TTP-ICG (n = 3). (*C*) CCK8 assay of TTP-ICG on MDA-MB-231-Luc cells, MCF-7 cells, and MCF-10A cells (n = 5).

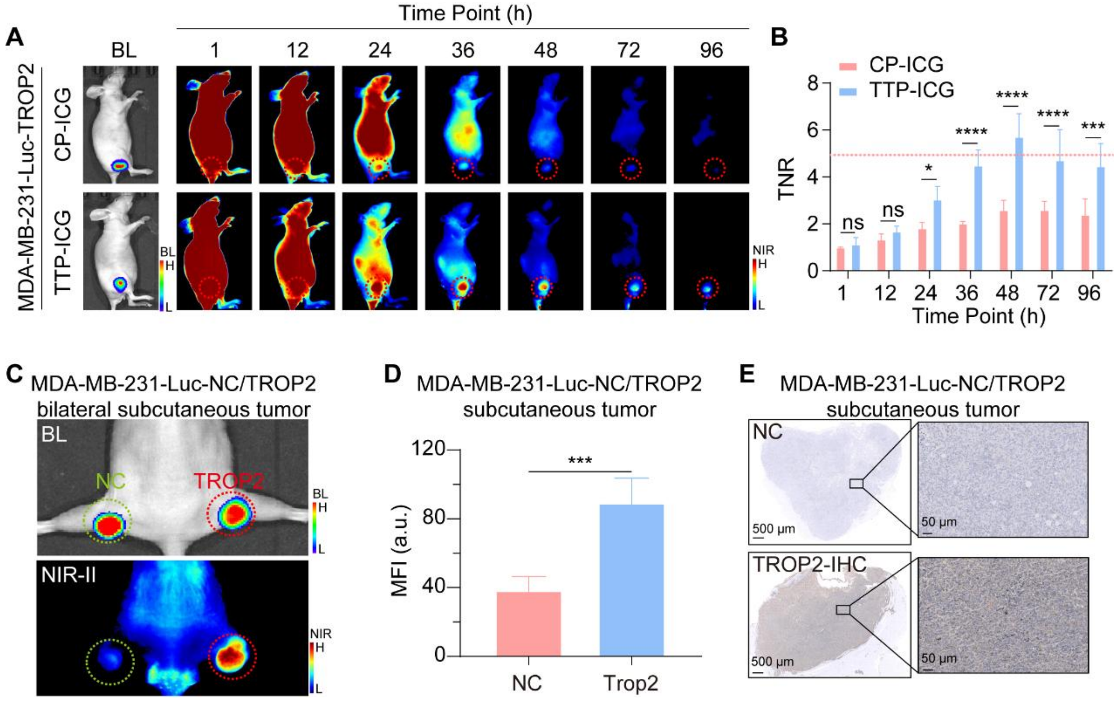

Fig. S6. TROP2-targeting validation of TTP-ICG in subcutaneous mouse models. (*A* and *B*) Representative *in vivo* bioluminescence (BL) and NIR-II images (*A*) and TNRs (*B*) analysis after injection of CP-ICG/TTP-ICG in MDA-MB-231-Luc-TROP2 tumor-bearing mice. Red dotted line in B is according to the Rose criterion. (*C*-*E*) Representative BL and NIR-II images (*C*), tumor NIR-II MFI analysis (*D*) and TROP2-immunohistochemistry (IHC) result (*E)* at 48 h post-injection of TTP-ICG in bilateral tumor model of MDA-MB-231-Luc-NC and MDA-MB-231-Luc-TROP2. (* $P < 0.05$, *** $P < 0.001$, **** $P < 0.0001$, mean ± SD, n = 4)

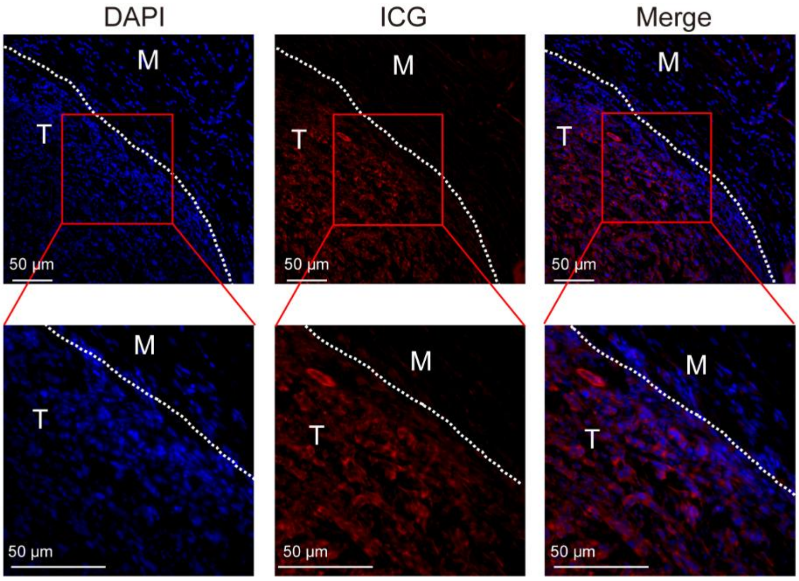

Fig. S7. TROP2-targeting in frozen tumor-muscle sections. Representative fluorescence microscopy images after incubating frozen section of tumor with TTP-ICG. White dotted line indicates the boundary between tumor and muscle.

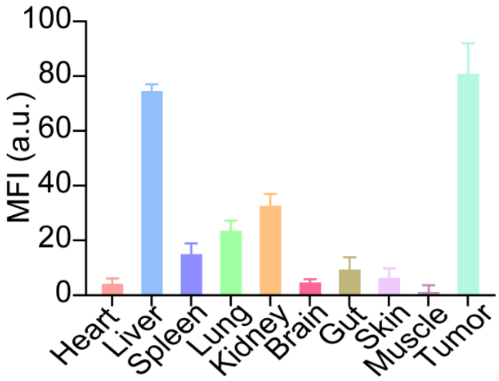

Fig. S8. NIR-II MFI analysis for biodistribution of TTP-ICG at 48 h post-injection in 4T1-Luc-TROP2 subcutaneous tumor model. (n = 4)

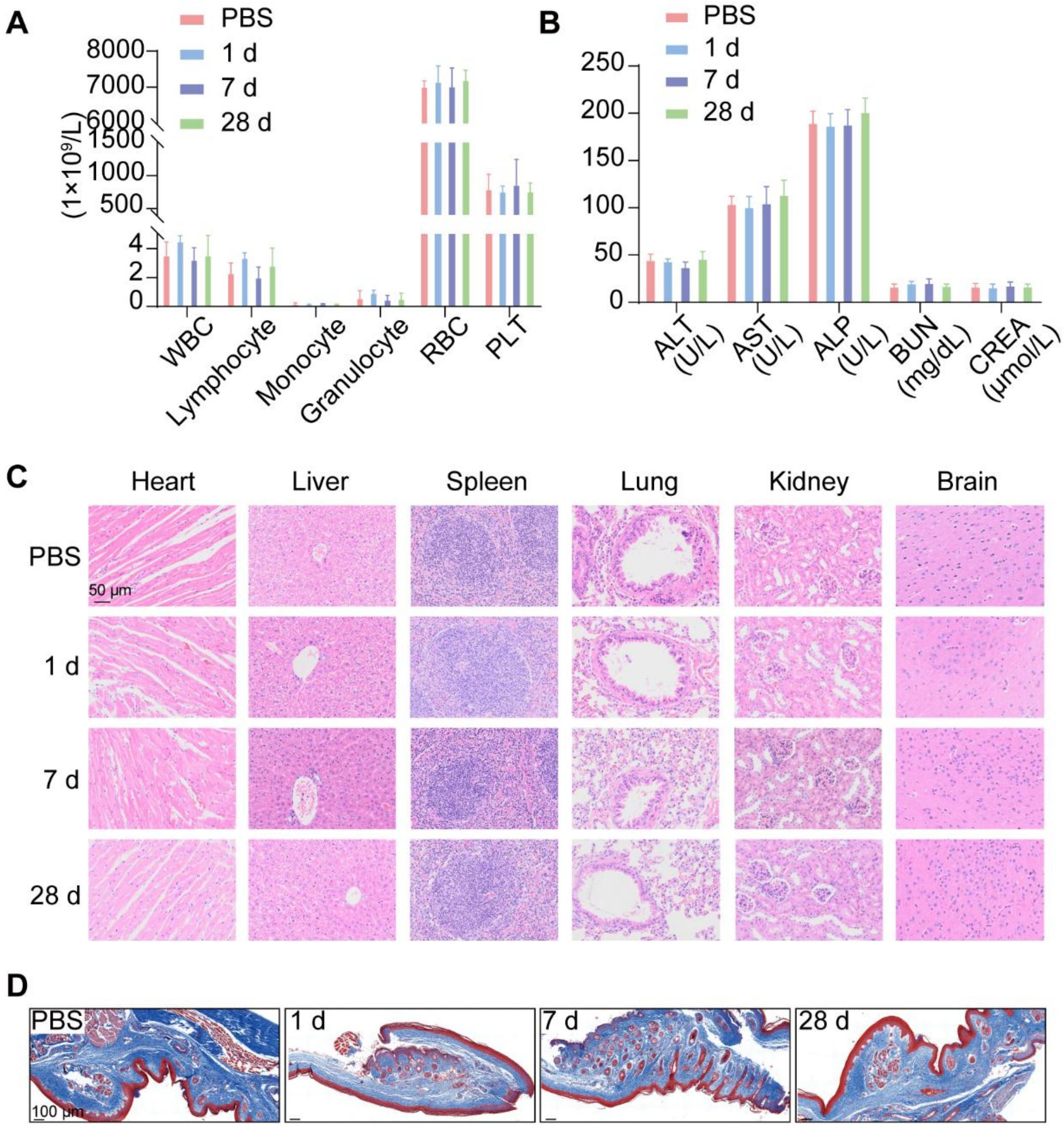

Fig. S9. Biocompatibility validation of TTP-ICG in BALB/c mice. (*A* and *B*) Blood routine (*A*) and blood biochemistry (*B*) results of BALB/c mice at 1, 7, and 28 days after intravenous injection of TTP-ICG, as well as an equivalent volume of PBS. (mean ± SD, n = 5). (*C*) Representative H&E staining results of vital organs from BALB/c mice at 1, 7, and 28 days after tail vein injection of TTP-ICG and an equivalent volume of PBS. The scale bar represents 50 μm. (*D*) Representative Masson staining results of skin tissues from foot pads after subcutaneous injection of TTP-ICG. The scale bar represents 100 μm.

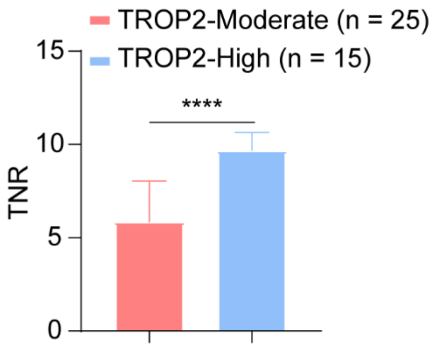

Fig. S10. TNR analysis of tumors with different TROP2 expression (TROP2-moderate, H-Score 100-200; TROP2-high H-Score 200-300) in MMTV-PyVT mouse model. (**** $P <$ 0.0001, mean ± SD)

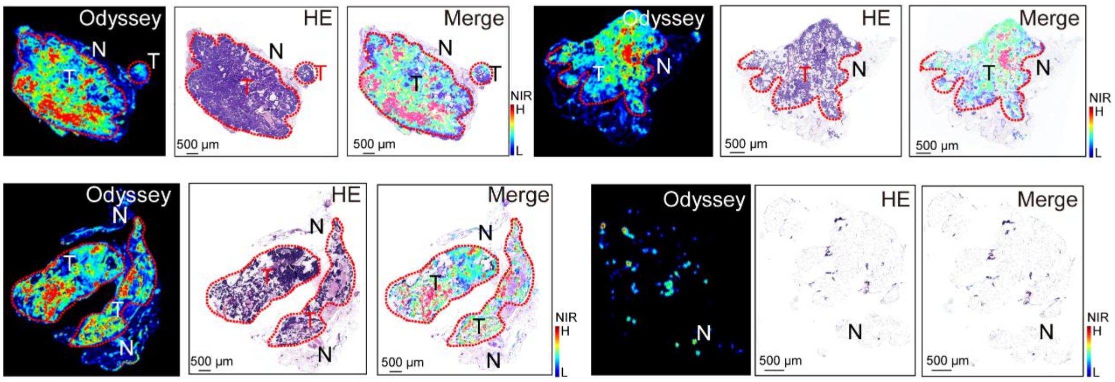

Fig. S11. Fluorescence and pathology colocalization analyses. Representative fluorescence and pathology colocalization analyses of tumor and normal tissues. The scale bar represents 500 μm.

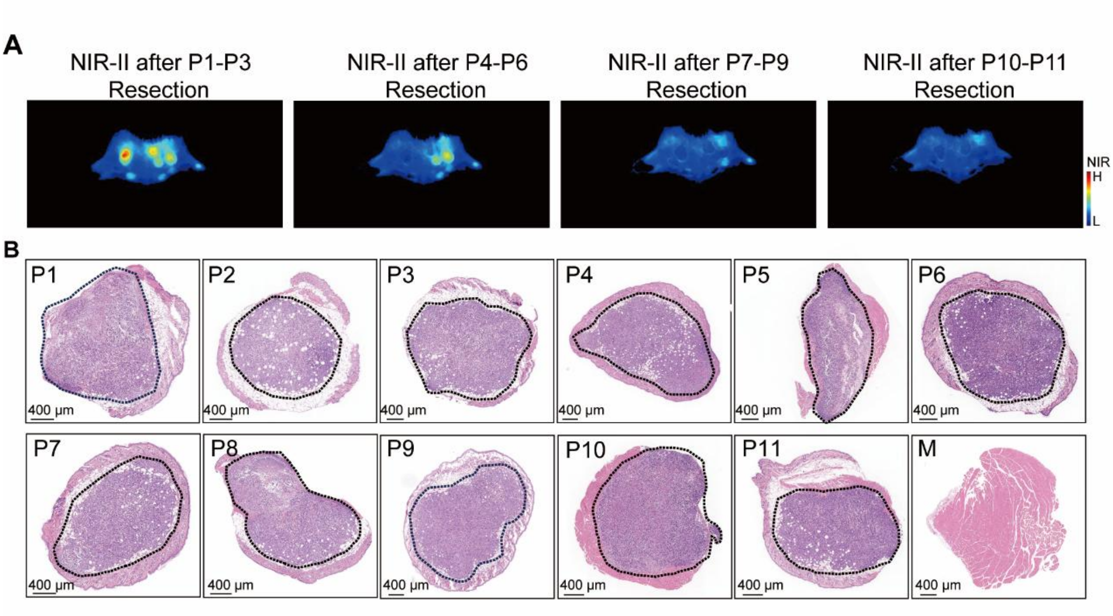

Fig. S12. NIR-II fluorescence-guided sequential resection of micro-tumors. (*A*) Representative procedure for sequential resection of micro-tumors from Fig. 3*F* under NIR-II fluorescence guidance. (*B*) H&E staining results of the resected tissues in (A). All scale bars represent 400 μm.

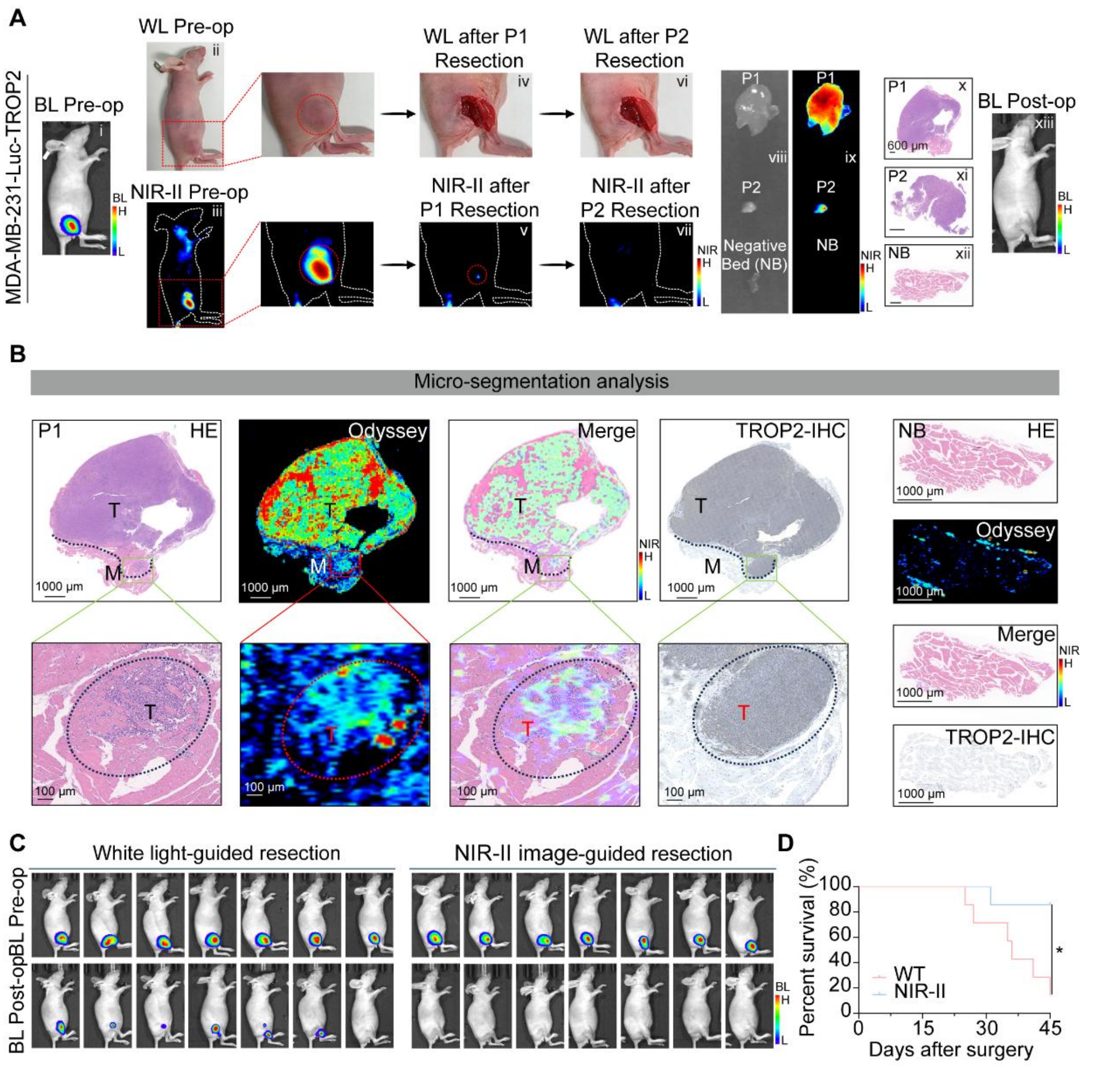

Fig. S13. NIR-II fluorescence-guided resection in the MDA-MB-231-Luc-TROP2 intramuscular tumor-invasion model. (*A*) Representative procedure for NIR-II fluorescence-guided resection in the MDA-MB-231-Luc-TROP2 intramuscular tumor-invasion model. All scale bars represent 600 μm. (*B*) The micro-segmentation fluorescence and pathological analyses of piece 1 and the negative bed in (A). All scale bars represent 1000 μm. (*C* and *D*) Recurrence (*C*) and survival (*D*) after tumor resection in the MDA-MB-231-Luc-TROP2 tumor-invasion model. (* $P < 0.05$, mean ± SD, n = 7)

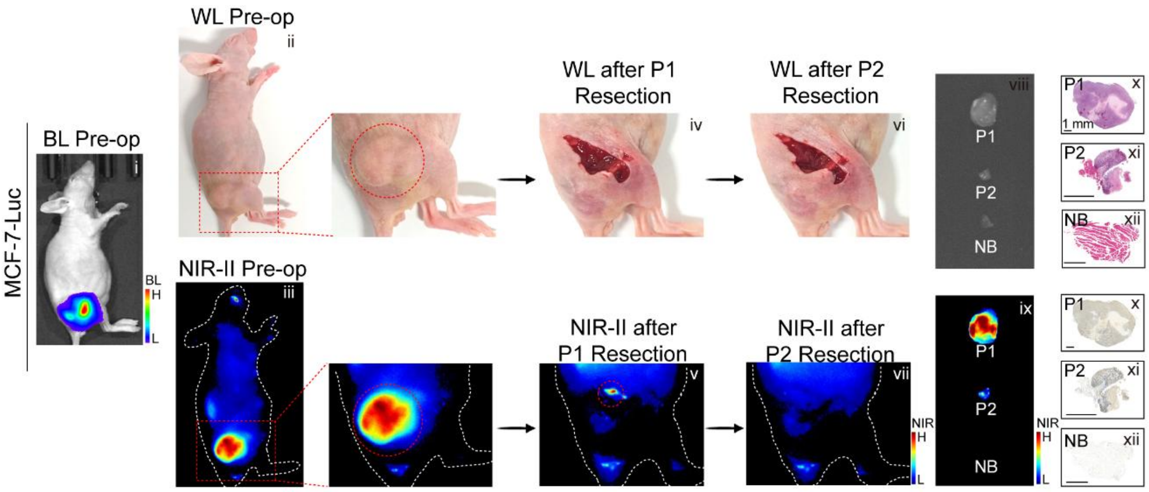

Fig. S14. Representative procedure for NIR-II fluorescence-guided resection in the MCF-7-Luc intramuscular tumor-invasion model. All scale bars represent 1 mm.

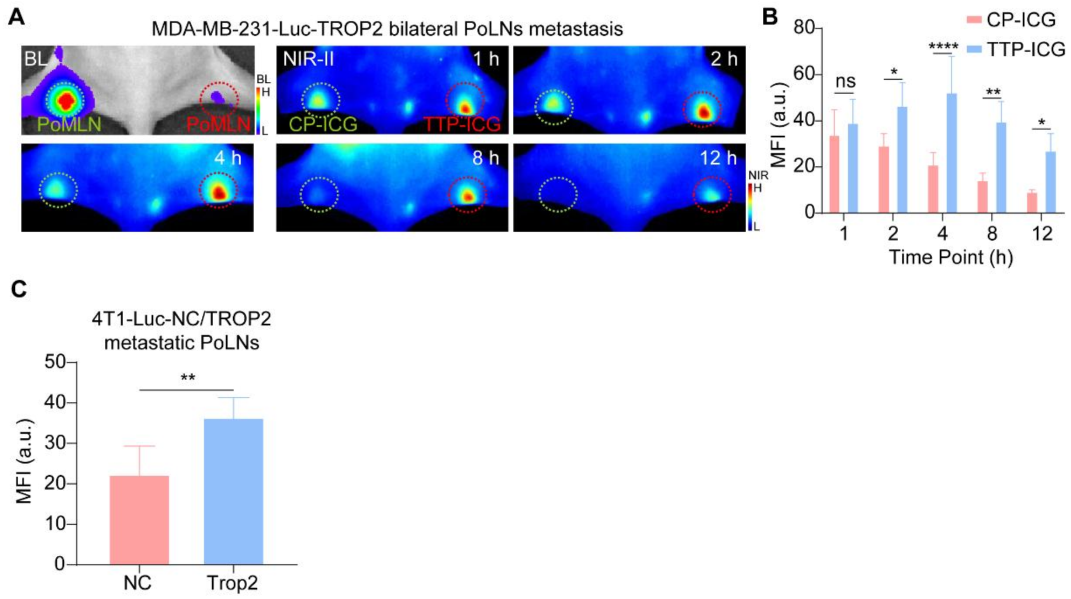

Fig. S15. TROP2-targeting validation in bilateral PoLN metastasis models. (*A* and *B*) Representative *in vivo* BL and NIR-II images (*A*) as well as NIR-II MFI analysis (*B*) of PoLNs after subcutaneous injection of CP-ICG or TTP-ICG via bilateral foot pads in MDA-MB-231-Luc-TROP2 bilateral PoLN metastasis model. (*C*) NIR-II MFI analysis of bilateral PoLNs from Fig. 4*C* at 12 h postinjection of TTP-ICG in bilateral PoLN metastasis model with 4T1-Luc-NC and 4T1-Luc-TROP2. (* $P < 0.05$, ** $P < 0.01$, **** $P < 0.0001$, mean ± SD, n = 4)

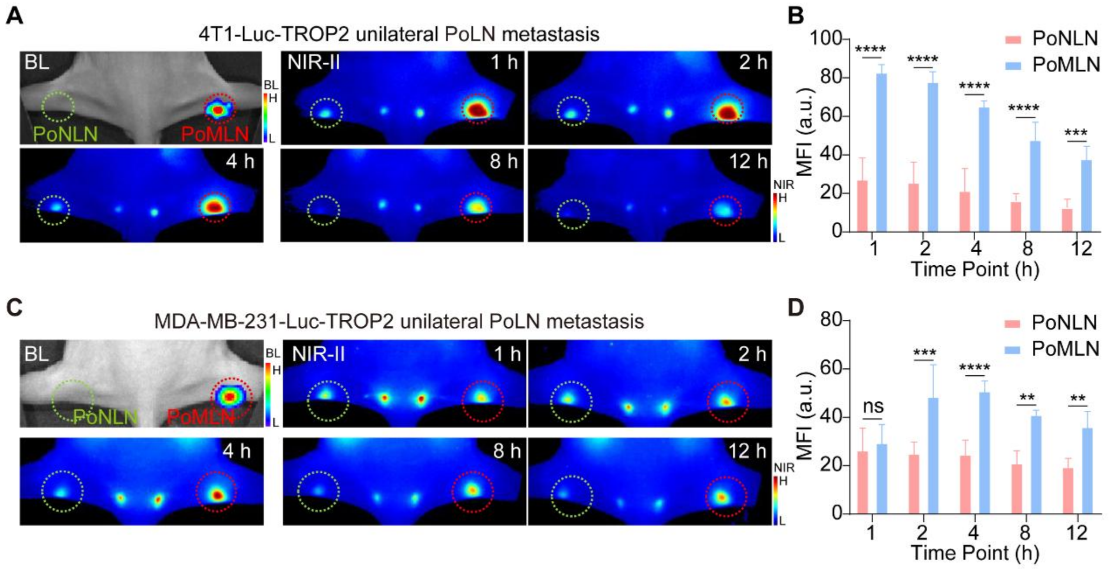

Fig. S16. TROP2-targeting validation in unilateral PoLNs metastasis models. Representative *in vivo* BL and NIR-II images (*A* and *C*) as well as NIR-II MFI analysis (*B* and *D*) of non-metastatic PoLNs (PoNLNs) and metastatic PoLNs (PoMLNs) after subcutaneous injection of TTP-ICG via bilateral foot pads in 4T1-Luc-TROP2 (up) and MDA-MB-231-Luc-TROP2 (down) unilateral PoLN metastasis model. (** $P < 0.01$, *** $P < 0.001$, **** $P < 0.0001$, mean ± SD, n = 4)

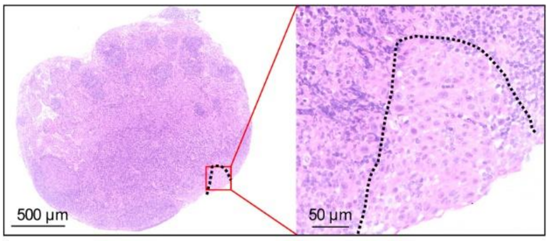

Fig. S17. H&E staining result of right SaLN from 4T1-Luc-TROP2 unilateral PoLN metastasis model at 25 days post-molding in Fig. 4*K*.

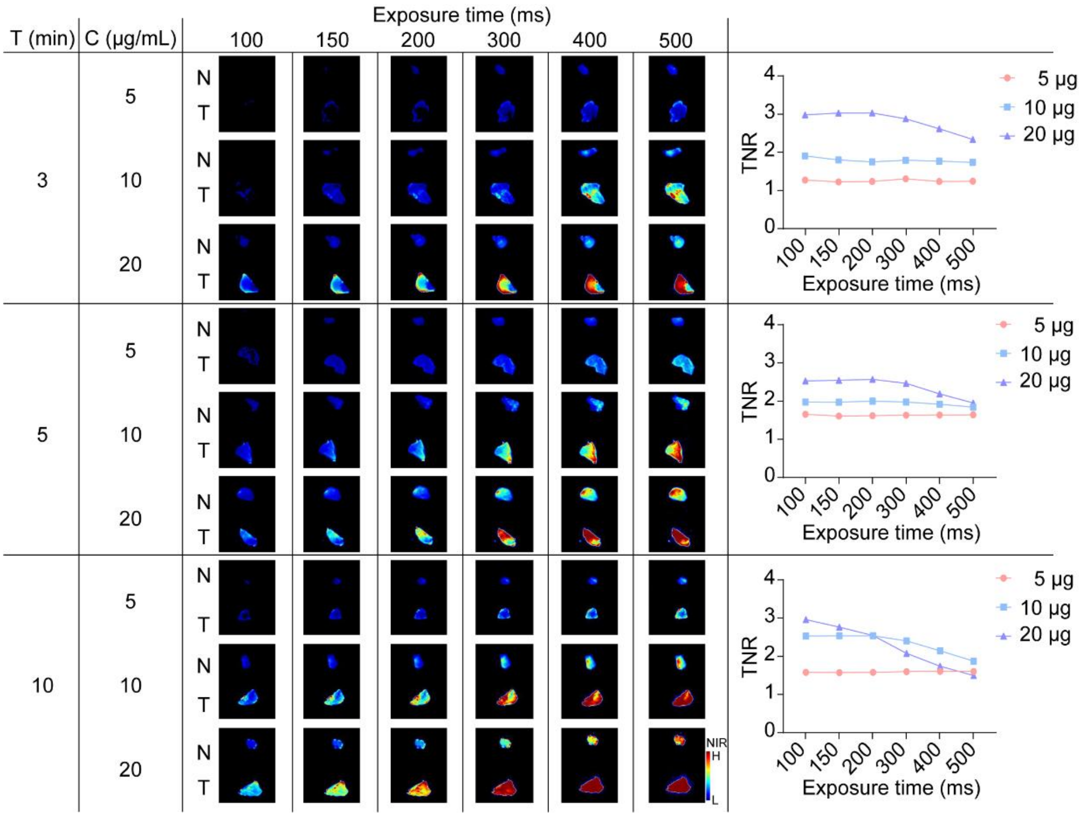

Fig. S18. NIR-II images and TNR analysis of normal mammary and 4T1-Luc-TROP2 cancer tissues after RIIM. Representative *ex vivo* NIR-II images and TNR analysis of normal mammary gland and 4T1-Luc-TROP2 cancer tissues from mouse model after TTP-ICG-based RIIM with different incubation times (3, 5, 10 min) and TTP-ICG concentrations (5, 10, 20 μg/mL) under different exposure time (100, 150, 200, 300, 400, 500 ms). (n = 4).

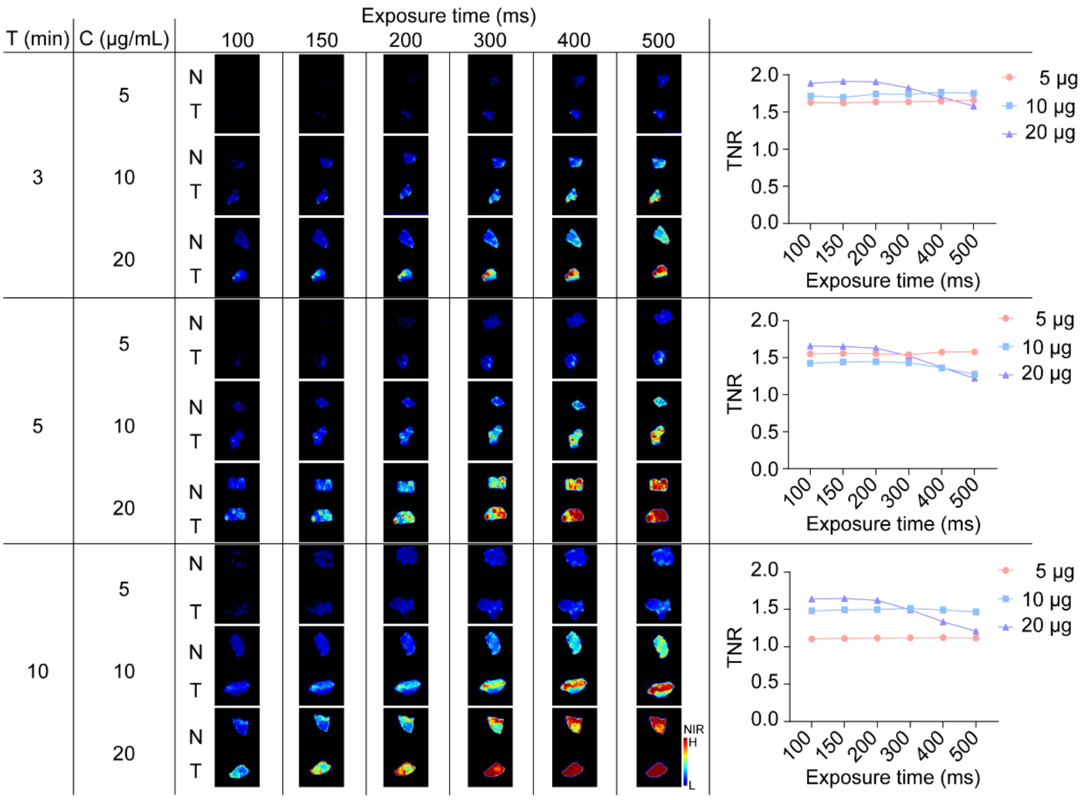

Fig. S19. NIR-II images and TNR analysis of patients' normal mammary and breast cancer tissues after RIIM. Representative *ex vivo* NIR-II images and TNR analysis of normal mammary gland and breast cancer tissues from patients after TTP-ICG-based RIIM with different incubation times (3, 5, 10 min) and TTP-ICG concentrations (5, 10, 20 μg/mL) under different exposure time (100, 150, 200, 300, 400, 500 ms). (n = 4).

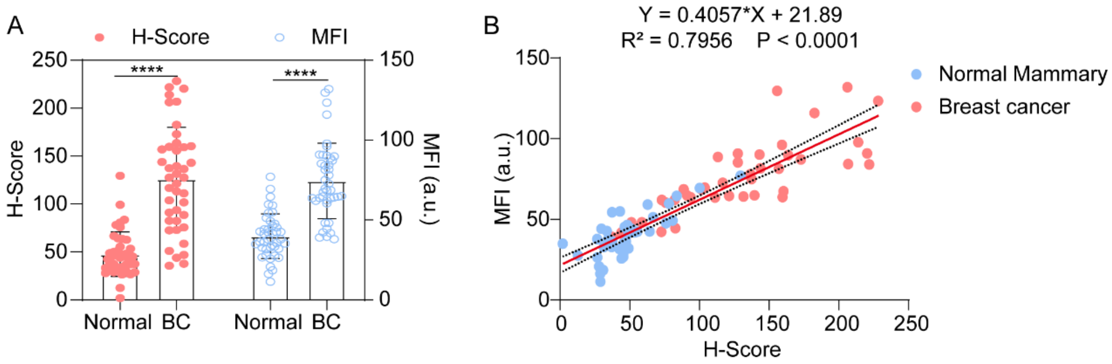

Fig. S20. Correlation analysis between MFI and the expression of TROP2 in BC tissues determined by RIIM. (*A*) H-Score (TROP2) and MFI of normal mammary gland and BC tissues treated by RIIM. (*B*) Linear regression analysis of H-Score (TROP2) and MFI. (n = 42, **** $P < 0.0001$, mean ± SD)

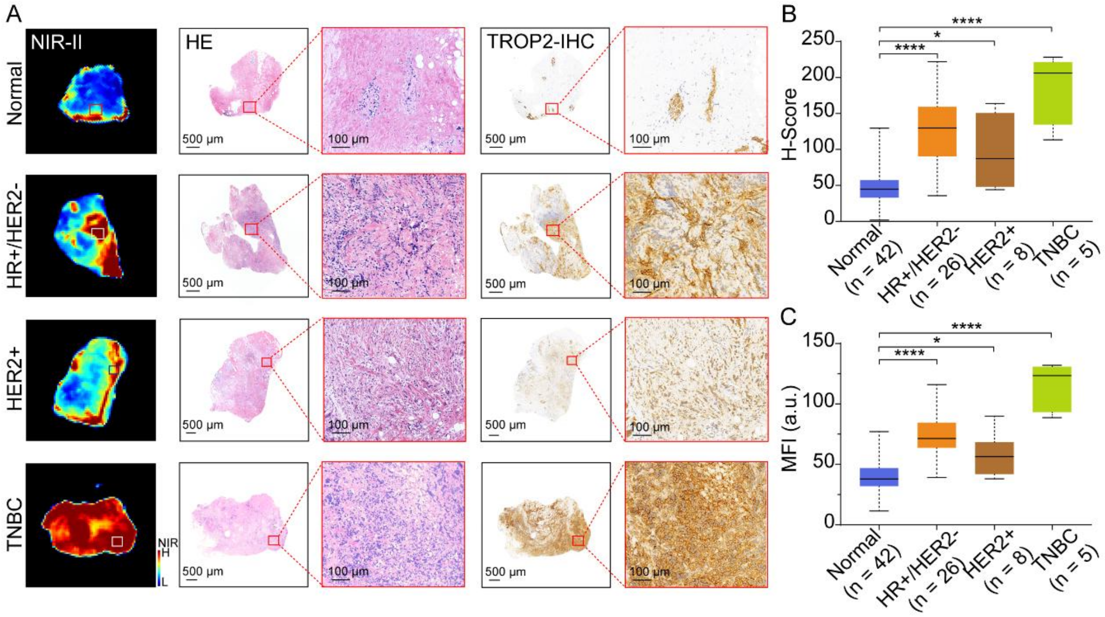

Fig. S21. TROP2 expression levels and MFI assessment across different subtypes of breast cancer after detected with RIIM. (*A*) Representative ex vivo NIR-II images and pathological analyses of normal mammary gland and BC tissues with different subtypes (HR+/HER2-, HER2+ and TNBC) after RIIM. (*B*) TROP2 expression levels of normal mammary gland and BC samples with different subtypes. (*C*) MFI of normal mammary gland and BC samples with different subtypes assessed using RIIM. (* $P < 0.05$, **** $P < 0.0001$, mean ± SD)

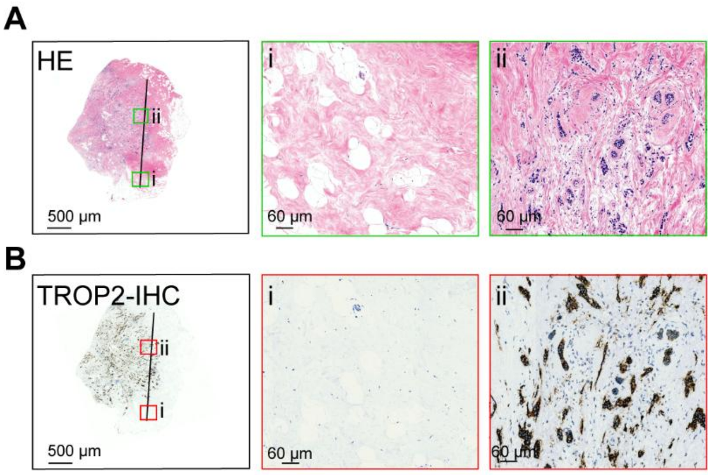

Fig. S22. The H&E staining (*A*) and TROP2-IHC (*B*) staining result of the breast cancer tissue from Fig. 5*H*. The i indicates the normal tissue and the ii indicates the malignant tissue in the IOL.

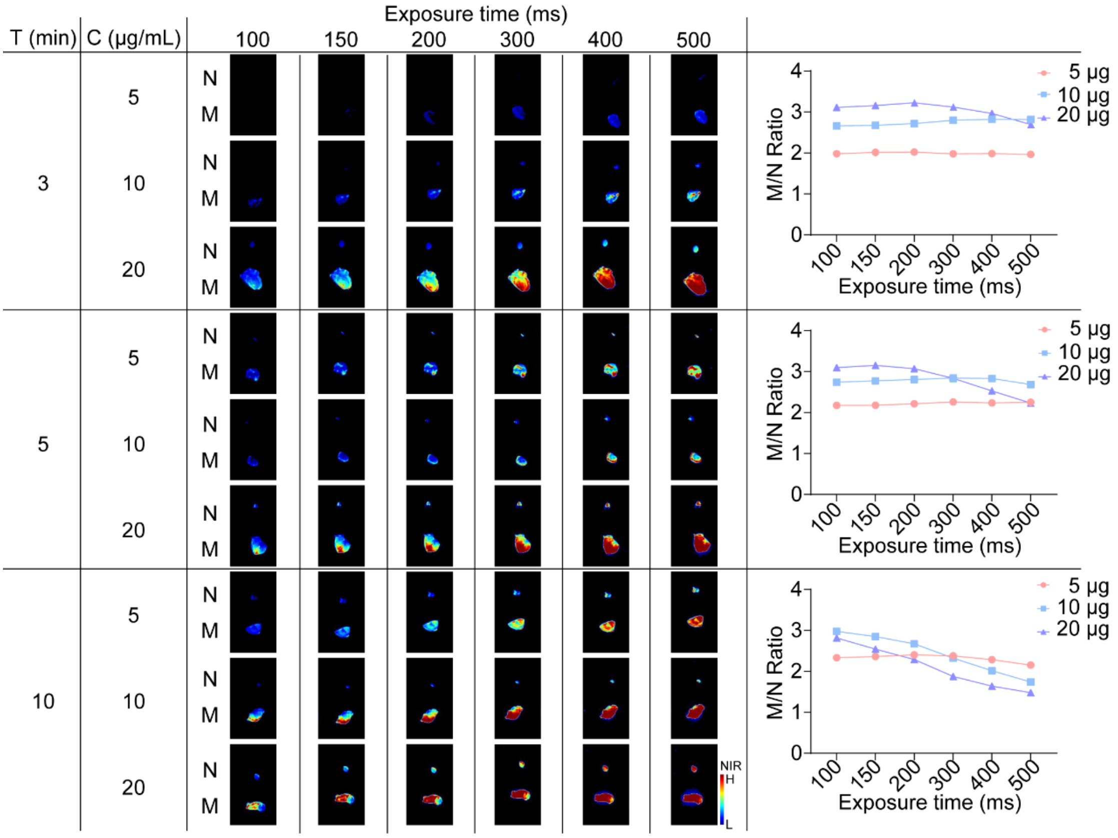

Fig. S23. NIR-II images and TNR analysis of normal LNs and 4T1-Luc-TROP2 metastatic LNs tissues after RIIM. Representative *ex vivo* NIR-II images and TNR analysis of normal LNs and 4T1-Luc-TROP2 metastatic LNs tissues from mouse model after TTP-ICG-based RIIM with different incubation times (3, 5, 10 min) and TTP-ICG concentrations (5, 10, 20 μg/mL) under different exposure time (100, 150, 200, 300, 400, 500 ms). (n = 4).

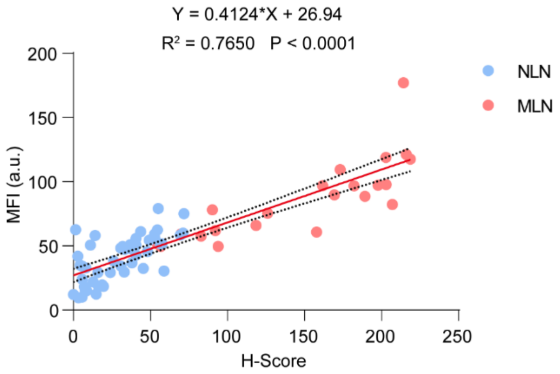

Fig. S24. Correlation analysis of the MFI and the expression of TROP2 in lymph nodes determined by RIIM.

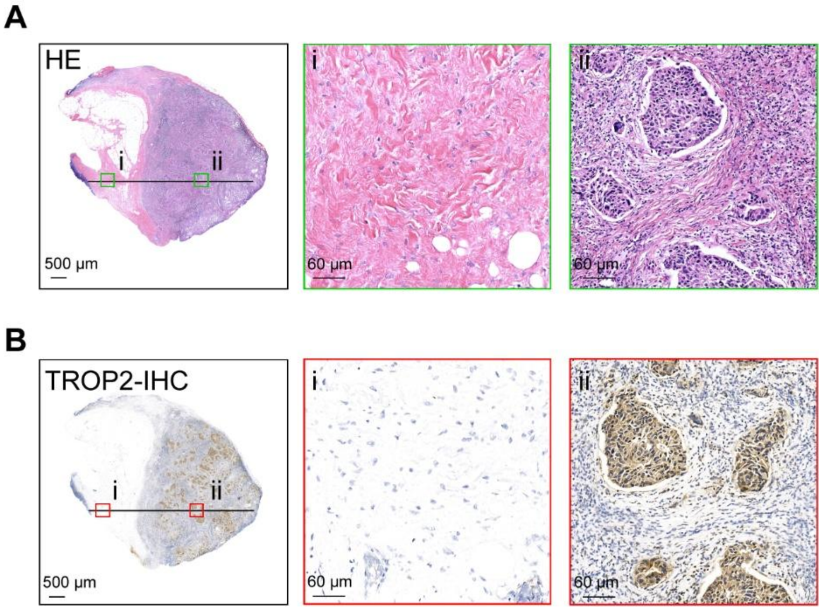

Fig. S25. The H&E staining (*A*) and TROP2-IHC (*B*) staining result of the MLN from Fig. 6*F*. The i indicates the normal tissue and the ii indicates the metastatic lesion in the IOL.

Tables

Table S1. Baseline characteristics of breast cancer patients enrolled in this study.

**Characteristic**	**Value**
**Age (years)**	53.98 ± 11.62
**Race**	Asian: 48 (100%)
Ki-67 (%)	
< 20	12 (25%)
20 - 30	13 (27.08%)
> 30	23 (47.92%)
**Pathology**	
DCIS	5 (10.42%)
IDC	39 (81.25%)
DCIS+IDC	2 (4.17%)
ILC	2 (4.17%)
**Molecular subtype**	
HR+/HER2-	29 (60.42%)
HER2+	9 (18.75%)
TNBC	5 (10.42%)
**Neoadjuvant**	10 (20.83%)
**Non-neoadjuvant**	38 (79.17%)

Notes: DCIS, ductal carcinoma in situ; IDC, invasive ductal carcinoma; ILC, invasive lobular carcinoma; HER2, human epidermal growth factor receptor 2; TNBC, triple-negative breast cancer. Data presented as means ± SD or number of participants (% of cohort). *n*=48

Table S2. Diagnostic performance of RIIM (cutoff = 60.12) in identifying breast cancers using pathology assessment as a standard method.

	**Pathology**	
**RIIM**	Positive (n)	Negative (n)
Positive	34	3
Negative	8	39

Table S3. Diagnostic performance of RIIM (cutoff = 59.14) in identifying metastatic lymph nodes using pathology assessment as a standard method.

	**Pathology**	
**RIIM**	Positive (n)	Negative (n)
Positive	18	6
Negative	4	43

SI References

1. R. Q. Yang, *et al.*, Development and Preclinical Evaluation of a Near-Infrared Fluorescence Probe Based on Tailored Hepatitis B Core Particles for Imaging-Guided Surgery in Breast Cancer. *Int. J. Nanomedicine* 17, 1343-1360 (2022).
